# Genome assembly and resequencing shed light on evolution, population selection, and sex identification in *Vernicia montana*

**DOI:** 10.1093/hr/uhae141

**Published:** 2024-05-18

**Authors:** Wenying Li, Xiang Dong, Xingtan Zhang, Jie Cao, Meilan Liu, Xu Zhou, Hongxu Long, Heping Cao, Hai Lin, Lin Zhang

**Affiliations:** Key Laboratory of Cultivation and Protection for Non-Wood Forest Trees of the Ministry of Education and Key Laboratory of Non-Wood Forest Products of the Forestry Ministry, Central South University of Forestry and Technology, Shaoshan South Road, No.498, Tianxin District, Changsha, Hunan 410004, China; College of Biology and Agricultural Resources, Huanggang Normal University, No.146 Xingang 2nd Road, Huangzhou District, Huanggang, Hubei 438000, China; Key Laboratory of Cultivation and Protection for Non-Wood Forest Trees of the Ministry of Education and Key Laboratory of Non-Wood Forest Products of the Forestry Ministry, Central South University of Forestry and Technology, Shaoshan South Road, No.498, Tianxin District, Changsha, Hunan 410004, China; Shenzhen Branch, Guangdong Laboratory for Lingnan Modern Agriculture, Genome Analysis Laboratory of the Ministry of Agriculture, Agricultural Genomics Institute at Shenzhen, Chinese Academy of Agricultural Sciences, No.7 Pengfei Road, Dapeng New District, Shenzhen 518120, China; Key Laboratory of Cultivation and Protection for Non-Wood Forest Trees of the Ministry of Education and Key Laboratory of Non-Wood Forest Products of the Forestry Ministry, Central South University of Forestry and Technology, Shaoshan South Road, No.498, Tianxin District, Changsha, Hunan 410004, China; Key Laboratory of Cultivation and Protection for Non-Wood Forest Trees of the Ministry of Education and Key Laboratory of Non-Wood Forest Products of the Forestry Ministry, Central South University of Forestry and Technology, Shaoshan South Road, No.498, Tianxin District, Changsha, Hunan 410004, China; College of Landscape Architecture, Central South University of Forestry and Technology, Shaoshan South Road, No.498, Tianxin District, Changsha, Hunan 410004, China; Key Laboratory of Cultivation and Protection for Non-Wood Forest Trees of the Ministry of Education and Key Laboratory of Non-Wood Forest Products of the Forestry Ministry, Central South University of Forestry and Technology, Shaoshan South Road, No.498, Tianxin District, Changsha, Hunan 410004, China; U.S. Department of Agriculture, Agricultural Research Service, Southern Regional Research Center, 1100 Allen Toussaint Blvd, New Orleans, LA 70124-4305, USA; Key Laboratory of Cultivation and Protection for Non-Wood Forest Trees of the Ministry of Education and Key Laboratory of Non-Wood Forest Products of the Forestry Ministry, Central South University of Forestry and Technology, Shaoshan South Road, No.498, Tianxin District, Changsha, Hunan 410004, China; Key Laboratory of Cultivation and Protection for Non-Wood Forest Trees of the Ministry of Education and Key Laboratory of Non-Wood Forest Products of the Forestry Ministry, Central South University of Forestry and Technology, Shaoshan South Road, No.498, Tianxin District, Changsha, Hunan 410004, China

## Abstract

*Vernicia montana* is a dioecious plant widely cultivated for high-quality tung oil production and ornamental purposes in the Euphorbiaceae family. The lack of genomic information has severely hindered molecular breeding for genetic improvement and early sex identification in *V. montana*. Here, we present a chromosome-level reference genome of a male *V. montana* with a total size of 1.29 Gb and a contig N50 of 3.69 Mb. Genome analysis revealed that different repeat lineages drove the expansion of genome size. The model of chromosome evolution in the Euphorbiaceae family suggests that polyploidization-induced genomic structural variation reshaped the chromosome structure, giving rise to the diverse modern chromosomes. Based on whole-genome resequencing data and analyses of selective sweep and genetic diversity, several genes associated with stress resistance and flavonoid synthesis such as CYP450 genes and members of the LRR–RLK family, were identified and presumed to have been selected during the evolutionary process. Genome-wide association studies were conducted and a putative sex-linked insertion and deletion (InDel) (Chr 2: 102 799 917-102 799 933 bp) was identified and developed as a polymorphic molecular marker capable of effectively detecting the gender of *V. montana*. This InDel is located in the second intron of *VmBASS4*, suggesting a possible role of *VmBASS4* in sex determination in *V. montana.* This study sheds light on the genome evolution and sex identification of *V. montana*, which will facilitate research on the development of agronomically important traits and genomics-assisted breeding.

## Introduction

*Vernicia montana* and *Vernicia fordii* are the two most representative tree species of the genus *Vernicia* in the Euphorbiaceae family*.* Both species are commonly referred to as ‘tung oil trees’ or ‘tung trees’ and are economically important for the production of high-value tung oil from seeds and have important ornamental values in landscaping, due to their flower-related traits such as flower color, aroma, and abundant inflorescence phenotypes (Supplementary Data Fig. S1). Tung oil consists of approximately 80% α-eleostearic acid, which is highly susceptible to oxidation due to its three conjugated double bonds, and is thus widely used in various industrial fields, such as protective waterproof finishes, electrical insulating materials, environmentally friendly ink, biodiesel, etc. [1, 2]. In comparison with *V. fordii*, *V. montana* is a dioecious plant with female trees having higher fruit yields, and male trees displaying higher ornamental values due to large inflorescences. *V. montana* has been introduced to America, Argentina, Australia, Southeast Asia (e.g. Vietnam, Burma, and Laos), and East Asia (Japan and Korea) from southern China since the Qing dynasty for tung oil production [3] (Supplementary Data Fig. S2).

The *V. montana* stands as a cryptic dioecious species with a 1:1 sex segregation ratio in natural populations [4]. However, determining the plant sex of a non-flowering *V. montana* tree is difficult due to the lack of effective genomic methods for sex identification at present. A *V. montana* tree typically takes about 3 to 4 years to reach full maturity. Therefore, there is a need to develop an accurate, rapid, and effective method to determine the sex of *V. montana* seedlings in breeding programs. High-quality reference genomes and population-based genome resequencing data provide a solid data foundation for constructing plant genetic variant databases. Based on the genetic variants associated with different sexes, sex-specific variants determining the plant sex have been identified in some dioecious plants. For example, through genomic approaches combined with transcriptome analysis, two Y-encoded sex-determining candidate genes were identified as acting as the suppressor of feminization (*SyGl*) and maintenance of male functions (*FrBy*) in the male-specific region of the Y chromosome in kiwifruit, respectively [5, 6]. Besides plant sex research, a high-quality genome is crucial for elucidating the origin, evolution, and development of important traits in plants. Recently, the genome of *V. fordii* was reported to provide important insights into genome evolution and oil biosynthesis [7], but it is insufficient for use in *V. montana* due to the significant differences between the two species.

Although *V. montana* is a dioecious plant, little is known about its sex-determination system. In our previous studies, a hypothesis for the origin of dioecism from cosexuality via monoecism was proposed [4], suggesting a possible XY sex-determination system in *V. montana* [8, 9]. To this end, a male *V. montana* was sequenced and a chromosomal-level reference genome was presented using an integrated assembly approach of PacBio sequencing, a high-throughput chromosome conformation capture (Hi-C) procedure, and full-length transcripts in this study. The evolutionary dynamics of the *V. montana* genome were explored, including the phylogenetic position, whole genome duplication, and the burst of retrotransposons (RTs). Additionally, the population structure of 178 *V. montana* individuals from 10 major distribution areas in China was also analysed based on whole genome resequencing data. A putative sex-linked InDel was identified based on genome-wide association studies (GWAS) with sex traits of *V. montana* individuals, and then a polymorphic molecular marker was developed. This molecular marker allows for the identification of the sex of *V. montana* using a capillary electrophoresis (CE) platform. This study will shed light on genome evolution, population selection, and sex identification of *V. montana*; provide significant clues to unraveling the sex-determining system and identifying its sex-determining regions; and will promote genetic research for its breeding.

## Results

### Genome assembly and annotation

**Figure 1:**
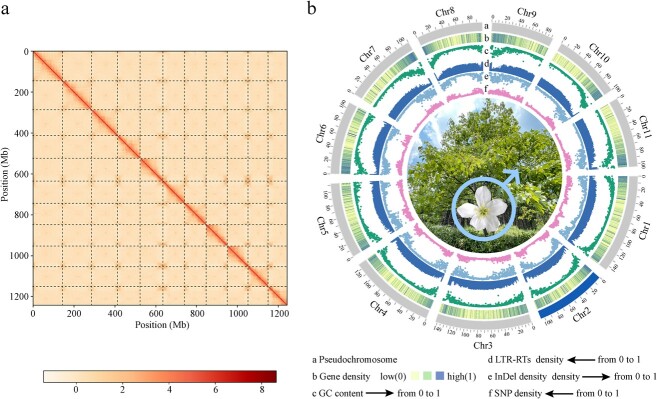
Hi-C-assisted assembly of *V. montana* pseudo-chromosomes. **a** The Hi-C interaction heatmap diagram at a resolution of 500 kb. **b** Genomic landscape of *V. montana*. All distributions are drawn using a window size of 2 Mb. Chr = Chromosome.

Based on the results of *K*-mer analysis (Supplementary Data Fig. S3), the estimated genome size of *V. montana* was 1.32 Gb with an expected heterozygosity of 0.39%. A total of 120.6 Gb of reads in CLR mode (about 92.6× coverage of the estimated sizes) were generated on the PacBio Sequel platform (Supplementary Data Tables S1 and S2). The genome was assembled *de novo* by combining PacBio long subreads, Hi-C maps, and Illumina short reads, resulting in a final assembly of 1.29 Gb, with a contig N50 of 3.69 Mb (Table 1). The completeness of the assembly of the *V. montana* genome was 97.2% (Supplementary Data Table S3). Eleven pseudo-chromosomes, numbered according to the corresponding homologous pseudo-chromosomes of *V. fordii* [7], with a size of 1.25 Gb, were anchored according to the Hi-C maps, which accounted for 97.28% of the assemblies (Fig. 1 and Table 1).

**Table 1:** Statistics for assembly and annotation of the *V. montana* genome

**Chromosome ID**	**No. of contigs**	**Length (bp)**
Chr1	385	146 937 770
Chr2	236	106 151 824
Chr3	491	142 838 282
Chr4	322	127 603 220
Chr5	338	112 539 824
Chr6	300	108 765 604
Chr7	340	110 345 422
Chr8	233	98 571 254
Chr9	330	92 819 287
Chr10	314	100 901 168
Chr11	231	104 584 184
Total No. of contigs		4411
Total length of contigs (Mb)		1287
Contig N50 (Mb)		3.69
Total No. of anchored contigs		3520
Total length of chromosome-level assembly (Mb)		1252
Anchor rate (%)		97.28
No. of protein-coding genes		28 781

The combination of *de novo*, homologous, and RNA-based predictions harbored a total of 28 781 predicted protein-coding genes, accounting for 94.3% of the BUSCO completeness in the *V. montana* genome (Table 1, Supplementary Data Table S4). The average sequence length of the identified protein-coding gene was 5872 bp and on average each gene contained ~5 exons with an average length of 235 bp. Compared with the homologous sequences and protein domains, approximately 92.24% of the protein-coding genes were functionally annotated (Supplementary Data Table S5). *De novo* prediction combined with homologous identification identified a total of 1.01 Gb transposable elements (TEs), representing 78.58% of the genome (Supplementary Data Table S6). The non-coding RNAs, including 105 miRNAs, 807 tRNAs, and 2828 rRNAs were also predicted in the *V. montana* genome (Supplementary Data Table S7).

### The phylogenetic position of *V. montana* and COM clade

**Figure 2:**
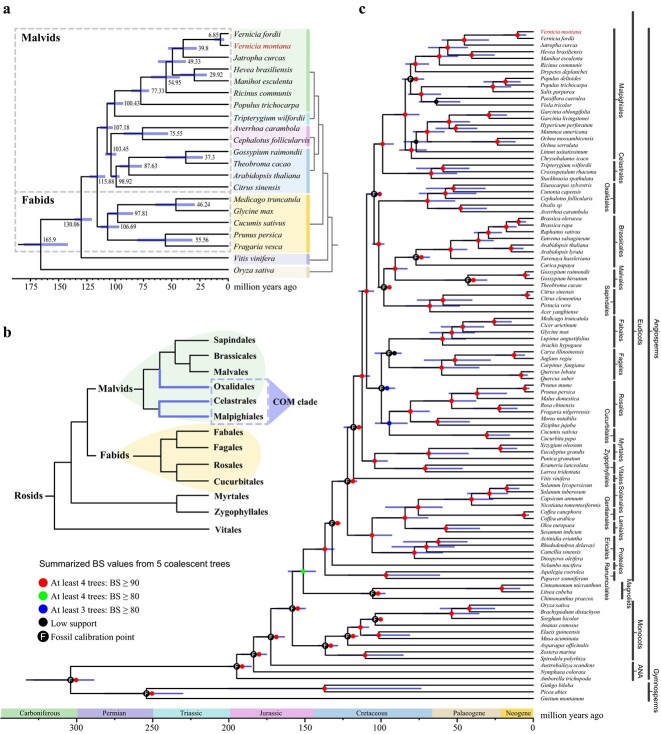
Phylogenetic relationships of angiosperms. **a** A summary of the phylogeny and timescale of 21 plant species based on the concatenated sequence alignments of the full nucleotide sequences of 141 single-copy nuclear genes. **b** The Phylogenetic relationship of 13 orders within the rosids. **c** A summary of the phylogeny and timescale of 101 plant species based on the coalescence-based nucleotide sequences of selected low-copy nuclear orthologous groups. The bars represent 95% credibility intervals for estimating divergence times.

Four concatenated and coalescent phylogenetic trees inferred from amino acid and nucleotide sequences of 141 orthologous single-copy nuclear genes from 21 species were constructed to investigate the evolution of *V. montana* and the Celastrales–Oxalidales–Malpighiales (COM) clade (Supplementary Data Fig. S4). The phylogenetic patterns of the concatenation-based gene trees inferred from amino acid and nucleotide sequences and the coalescent-based gene tree inferred from nucleotide sequences are consistent with each other, whereas the structure of the coalescent-based tree inferred from amino acid sequences is inconsistent (Supplementary Data Fig. S5). The concatenation-based tree inferred from nucleotide sequences was chosen as the phylogenetic relationship for the 21 species, thus estimating the divergence time of Euphorbiaceae species to be ~54.95 (42.81–68.36) Mya (Fig. 2a). The phylogenetic analysis confirms that *V. montana* is most closely related to *V. fordii*, which diverged from their common ancestor by about 6.85 (3.6–12.1) Mya in the Neogene period (Fig. 2a). All topologies agreed that the COM clade of species is affiliated with the malvids rather than the fabids. Precisely, the CM clade is parallel to the Oxalidales and other malvids (Fig. 2b). Moreover, the Zygophyllales and Myrtales, which were classified as sisters to the rest of the fabids and malvids, respectively, in the APG IV classification, were grouped and classified as sisters to the clade containing all the fabids and malvids (Fig. 2b). A summary of the phylogenetic patterns of low-copy nuclear (LCN) gene trees of 101 angiosperms also supports the structures of single-copy trees (Fig. 2c and Supplementary Data Figs S6–S10).

### Genome evolution following the core eudicot γ triplication

**Figure 3:**
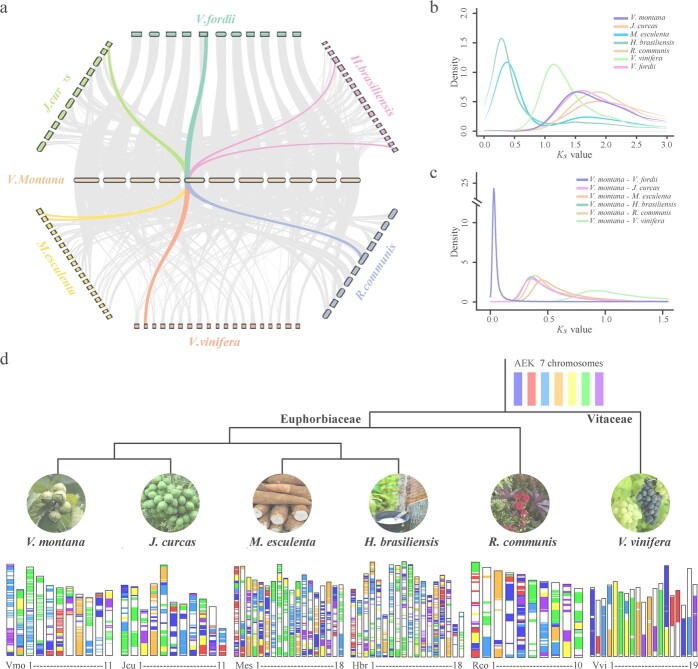
Comparative genomics of the Euphorbiaceae. **a** Syntenic relationships between the *V. montana* genomic regions and their putative orthologues present in *Vitis vinifera*, *Ricinus communis*, *Hevea brasiliensis*, *Manihot esculenta*, *Jatropha curcas*, and *Vernicia fordii*. **b**  *K_s_* distributions for paralogues found in the syntenic blocks of *V. montana* and *V. vinifera*. **c**  *K_s_* distributions for orthologues found in the syntenic blocks between *V. montana* and *V. vinifera*, *R. communis*, *H. brasiliensis*, *M. esculenta*, *J. curcas*, and *V. fordii*. **d** Model for the structural evolution of the *V. vinifera*, *R. communis*, *H. brasiliensis*, *M. esculenta*, *J. curcas*, *V. fordii*, and *V. montana* from the AEK ancestor (n = 7 chromosomes).

Pervasive polyploidy appears to have accelerated genome modifications and key innovations in land plant evolution [10]. Syntenic alignment of the *V. montana* genome with other Euphorbiaceae and *Vitis vinifera* demonstrated that *V. montana* did not undergo any recent whole genome duplication (WGD) after the ancient triplication (Fig. 3a and Supplementary Data Figs S11–S16). By conducting self–self synteny analysis, all syntenic block gene pairs were used to calculate the synonymous substitution rate (*K_s_*) values. The distribution of *K_s_* values showed another clear recent peak in *Manihot esculenta* and *Hevea brasiliensis*, but *V. montana* (~1.513) and the other species displayed only one peak above 1 (Fig. 3b), confirming that *V. montana* has experienced only one WGD event. Based on the distributions of *K_s_* between *V. montana* and other species, the peak values were all less than 1 (Fig. 3c), indicating that the WGD event of *V. montana* is older than its divergence from other species. Therefore, the WGD event of *V. montana* is an ancient γ event (whole genome triplication, WGT) shared by all eudicots, and the WGT of *V. montana* dates back to approximately 116.4 Mya. *V. vinifera* has always been used to discover ancestral traits and genomic features in flowering plants because it is believed that *V. vinifera* contains ancient genomic loci or ancestral gene orders [11]. To explore chromosomal evolution after the ancient triploidization event, we performed synteny analyses between *V. montana* and four other Euphorbiaceae species and *V. vinifera* using the pre-γ ancestral eudicot karyotype (AEK) (Fig. 3d). In contrast to *V. vinifera*, Euphorbiaceae species have undergone a large number of chromosomal rearrangement events during evolution and have retained many short syntenic blocks with the AEK. Among the Euphorbiaceae, *V. montana*, *Jatropha curcas*, and *Ricinus communis* (VJR) have experienced only the shared WGT event, whereas *M. esculenta* and *H. brasiliensis* (MH) have each experienced one more recent paleotetraploidy event, respectively. The VJR displayed a higher degree of structural conservation with the AEK compared to the MH. The model of chromosome evolution in Euphorbiaceae suggests that polyploidization-induced genomic structural variation reshaped chromosome structures, giving rise to diverse modern chromosomes since the common paleopolyploid ancestor.

### The recent burst of long terminal repeat retrotransposons (LTR-RTs) drives the genome size expansion of *V. montana*

**Figure 4:**
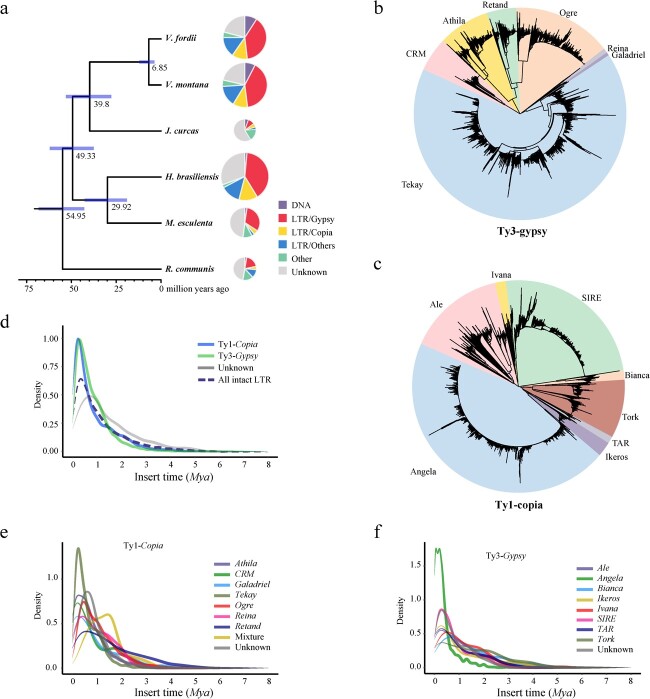
Evolution of RT families in the *V. montana* genome. **a** Proportion of different types of transposable elements (TE). **b** Phylogenetic relationship of Ty1-*Copia* LTR-RTs. **c** Phylogenetic relationship of Ty3-*Gypsy* LTR-RTs. **d**, **e,** and **f** Insertion time of different types of intact LTR-RTs.

The evolution of LTR-RTs and their potential contribution to the growth of the *V. montana* genome was investigated. The *V. montana* genome was enriched in RTs (855.8 Mb; 66.54%). LTR-RTs (60.96% Ty1-*Copia*, 16.10% Ty3-*Gypsy*, and 22.94% unknown) were particularly abundant, accounting for 66.34% of the genome size (853.2 Mb), which is nearly identical to the genomes of *V. fordii* (65.81%) and *H. brasiliensis* (65.88%) and is significantly higher than the genomes of *J. curcas* (18.39%), *M. esculenta* (39.72%), and *R. communis* (35.48%) (Fig. 4a, Supplementary Data Table S8). Intriguingly, the genome sizes of these plants were proportional to the proportions of LTR-RTs, and the TEs were largely determined by a small number of LTR-RTs families with extremely high copy numbers, e.g. the Ty3-*Gypsy* LTR-RTs (Fig. 4a). Ty3-*Gypsy* LTR-RTs families dominated the *V. montana* genome (520.16 Mb; 40.44%), with abundances almost 3.8 or 2.7 times higher than those of Ty1-*Copia* (137.33 Mb; 10.68%) or unknown LTR-RTs (195.73 Mb; 15.22%) (Fig. 4a, Supplementary Data Table S8).

In total, 15 998 intact LTR-RTs were identified, accounting for 11.57% (148.9 Mb) of the *V. montana* genome (Supplementary Data Table S9). The intact LTR-RTs were classified into 4753 families, of which the top 137 families with more than 10 copies comprised 67.2% of the intact LTR-RTs and 7.78% of the genome (Supplementary Data Table S9). Phylogenetic trees of intact Ty1-*Copia* and Ty3-*Gypsy* LTR-RTs were constructed, showing that the Ty1-*Copia* LTR-RTs are predominated by the *Angela* (41.77%) lineage followed by the *SIRE* (25%) lineage, whereas Ty3-*Gypsy* LTR-RTs are predominated by the *Tekay* (51.35%) lineage followed by the *Ogre* (22.39%) lineage (Fig. 4b and c, Supplementary Data Table S10). Analysis of intact LTR-RTs by the Kimura distance method [12] indicated that *V. montana* underwent multiple RT bursts caused by the amplification of Ty1-*Copia*, Ty3-*Gypsy*, and other classes of LTR-RTs (Fig. 4d–f). Grossly, the proliferation of LTR-RTs was concentrated in the last 1 Mya of the *V. montana* genome. The mean insertion time of all intact LTR-RTs was 0.3 Mya, with a very similar insertion time between Ty1-*Copia* (~0.23 Mya) and Ty3-*Gypsy* (~0.28 Mya) (Fig. 4d). Among the lineages in Ty1-*Copia*, two insertions were identified at 0.06 and 0.20 Mya in the *Angela* lineage, while the other lineages had insertions between 0.29 and 0.67 Mya (Fig. 4d). The majority of lineages in Ty3-*Gypsy* had insertions within 0.27 to 0.61 Mya.

### Population structure in *V. montana*

**Figure 5:**
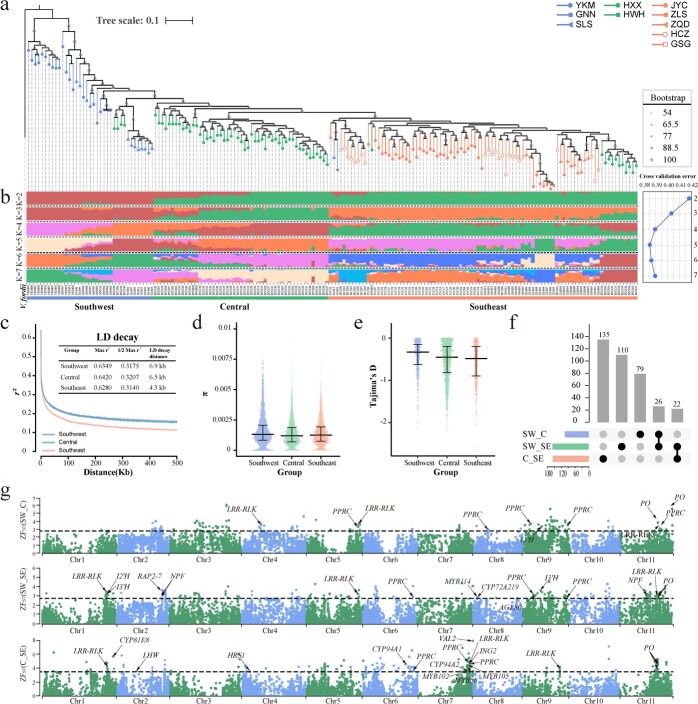
Population structure and genetic diversity analysis and gene identification in the selective sweeps. **a** Phylogenetic relationship, and **b** Population structure of 178 individuals. **c** Linkage disequilibrium (LD) decay. **d** Nucleotide diversity (π) ratios. **e** Tajima’s D values. **f** The upset plot of putative selective sweep genes between subgroup SW vs. Ce, SW vs. SE, and Ce vs. SE. **g** Several key genes related to stress resistance and flavonoid synthesis were identified in selective sweeps. The dashed lines represent the threshold of the top 1% of *F*_st_ for the Z-Score model (Z*F*_st_).

Relationships and divergence among 10 different ecological populations of 178 *V. montana* provenances were studied (Supplementary Data Fig. S17a). A total of 38 359 821 single-nucleotide polymorphisms (SNPs) and 4 266 202 insertion/deletions (InDels) were preliminarily identified. After hard filtering, a total of 10 166 492 high-quality SNPs with an average SNP frequency of about 7.9 per 1000 bp were obtained for further analysis. Using *V. fordii* as an outgroup, the phylogenetic relationships among the 178 accessions were explored based on the whole-genome genetic variations observed for single-copy genes. The neighbor-joining phylogenetic tree showed that the 178 accessions were mainly classified into three geographical subgroups, including (I) the Southwest China (SW) subgroup, consisting of 37 individuals mainly from Sichuan, Yunnan, and Guangxi provinces; (II) the Central China (Ce) subgroup, comprising 51 individuals from Hunan and Hubei provinces; and (III) the Southeast China (SE) subgroup, including 90 individuals mainly from Jiangxi, Zhejiang, Guangdong provinces (Fig. 5a). The principal component analysis (PCA) displayed similar population affinities (Supplementary Data Fig. S17b).

Ancestral population stratification was estimated for each accession using maximum likelihood estimation with ancestral population sizes K = 2–7 (Fig. 5b). At K = 2, the Ce and SE subgroups exhibited consistent genetic constitution but separated at K = 3, suggesting that the SE subgroup originated from the Ce subgroup (Fig. 5b). The best ancestry proportions (the distribution of K = 5 genetic clusters with minimal cross-validation error) displayed more complex genetic constitutions in these subgroups (Fig. 5b). Genetic constitution exhibit measurable regional similarities: accessions from similar ecological areas may have similar genotypes, possibly due to both natural selection and artificial selection. The Ce subgroup is primarily composed of HXX accessions and exhibits relatively clear boundaries with both the SW and SE subgroups. All five types of genetic clusters could be identified in HXX accessions, respectively grouped into SW subgroup (1 type), Ce subgroup (5 types), and SE subgroup (4 types) (Fig. 5b).

Linkage disequilibrium (LD), nucleotide diversity (π) ratios, and Tajima's D values in the population of *V. montana* were also studied based on SNPs within regions of single-copy genes. The LD statistic *r^2^* was used to estimate LD between pairwise comparisons of SNPs. The LD decayed to its half-maximum value at different rates: SE > Ce > SW (Fig. 5c). The relatively high LD decay rates in the SE subgroup may reflect its higher population genetic diversity. However, the SW subgroup had a higher average π ratio (0.00149 ± 0.001078) than the Ce subgroup (0.00135 ± 0.00108) and SE group (0.00139 ± 0.00109) (Fig. 5d). The negative Tajima's D values showed an increase (SE < Ce < SW), and the number of windows with negative was much higher (~2 fold) in the Ce subgroup than in the SW subgroup and SE subgroup (Fig. 5e), indicating a stronger positive selection in the Ce and SE subgroups than in the SW subgroup.

To identify reliable group-specific fixed genes by selective sweeps during migration, hybridization, and domestication, a combined strategy incorporating population divergence analysis (*F*_ST_) and π-ratio, which is calculated using SNPs within the coding regions of genes in a 100-kb sliding window and 10 kb step size was used. A total of 195 putative selective sweeps covering 29.36 Mb of the *V. montana* genome including 372 genes were identified through pairwise comparisons between different subgroups: SW vs. Ce, SW vs. SE, and Ce vs. SE (Supplementary Data Table S11). Functional enrichment based on Gene Ontology (GO) showed that these genes were mainly enriched for phosphatase activity, kinase activity, and DNA dephosphorylation (Supplementary Data Table S12). Most of these putative selective sweep genes are group-specific genes, and no common selective sweep genes were identified among SW, Ce, and SE, or specifically between Ce and SE (Fig. 5f). Adaptation is essential for the survival and reproduction of living organisms. Several stress resistance and flavonoid synthesis genes that overlap with each other in the different pairwise comparisons are located within putative sweep selective regions, such as the CYP450 family genes *CYP94A1*/*CYP81E8*/*CYP726A20*, LRR-RLK family members, the NRT1/PTR family proteins NPF2.2/2.3/2.6/7.1, and the *I2′H* and *I3′H*. (Fig. 5g).

### Genome-wide association analysis of sex traits in *V. montana*

Twenty-five males and 25 females with relatively different population stratifications were selected for the GWAS based on the results of the ancestral population stratification assessment (Supplementary Data Table S13). Based on a Genome-wide Efficient Mixed-Model Association algorithm, associations of polymorphisms with the sex trait of *V. montana* individuals were identified using SNPs and InDels for these 50 individuals, respectively. A total of 66 and 18 significantly sex-associated SNPs and InDels, all of which were located on Chr 2, were identified using the Genetic Type I error calculator [12] (Supplementary Data Fig. S18, Tables S14 and S15).

**Figure 6:**
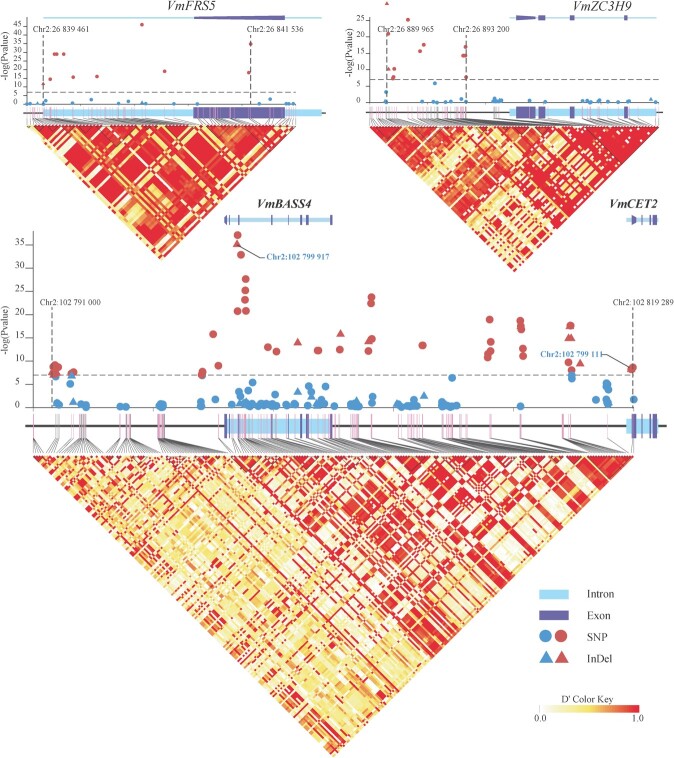
Linkage disequilibrium (LD) mapping of sex-associated alleles identified by genome-wide association studies. Red dots and triangles above the horizontal dashed lines indicate significant sex-associated SNPs and InDels (–logPvalue >7). Thin grey lines represent strands of chromosome 2. Pink bars on the grey lines of the chromosome represent already identified SNPs.

These sex-associated polymorphic loci were intensely distributed in three small regions of chromosome 2, covering 2.076, 3.291, and 28.29 kb regions, respectively, and were located in the promoter or gene region of four predicted protein-coding genes, including a FAR1-related sequence 5 (*FRS5*), a finger CCCH domain-containing protein 9 gene (*ZC3H9*), a sodium/metabolite cotransporter 4 (*BASS4*) and a CEN-like protein 2 gene (*CET2*) (Fig. 6). To explore the expression profile of these genes, the transcriptomics data of pistillate and staminate flowers at different developmental stages were used in this study. *VmFRS5* and *VmBASS4* exhibited extremely low expression levels in flowers (Supplementary Data Fig. S19). The *VmZC3H9* was expressed at all developmental stages in both pistillate and staminate flowers (Supplementary Data Fig. S19). The *VmCET2*, whose homologs are responsible for controlling inflorescence architecture and extending the phasing of shoot meristems in apple [13], kiwifruit [14], *Citrus* [15], is expressed only during the early flower developmental stages of staminate flowers (Supplementary Data Fig. S19), suggesting its potentially specific function for the early development of staminate flowers. A significant sex-associated SNP located at Chr 2: 102819111 (wald *p* value = 2.27e^−9^) resulted in a missense mutation of Leucine (GAT) to Asparagine (ATT) in approximately 64% (16 out of 25) of the females, as found in the fourth exon of *VmCET2* (Fig. 6).

**Figure 7:**
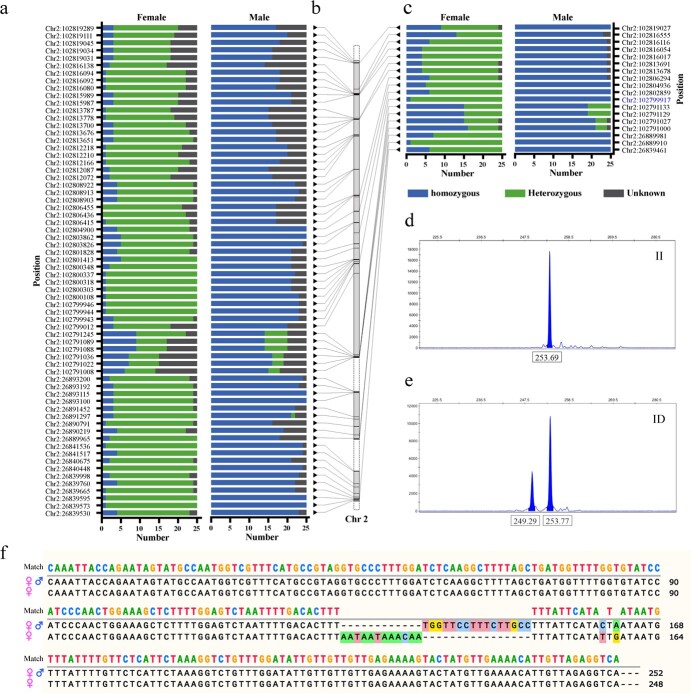
Identification of an InDel polymorphic molecular marker for sex identification of *V. montana*. **a** and **c** Genotypes of significant sex-associated SNPs and InDels obtained using GATK4. **b** Distribution of sex-associated SNPs and InDels on Chromosome 2. **d** and **e** Capillary electrophoresis of amplification products showing the insertion homozygote (II) and insertion/deletion heterozygote (ID) genotypes. f Alignment of fragments examined by Sanger sequencing.

In all selected male individuals, 59 of the 66 significantly sex-associated SNPs and 13 of the 16 significantly sex-associated InDels were genotypically homozygous, and the remaining significantly sex-associated loci in the majority of males also displayed homozygous genotypes (Fig. 7a–c, Supplementary Data Tables S14 and S15). For significantly sex-associated polymorphic loci in females, although the genotypes of most SNPs (64 out of 66) and InDels (13 out of 18) in more than half of the females were heterozygous, only 3 of 66 SNPs and 0 of 16 InDels displayed heterozygous genotypes in all selected females (Fig. 7a–c, Supplementary Data Tables S14 and S15). Briefly, the sex-associated SNPs and InDels were almost homozygous in males, whereas they tended to be heterozygous in females.

To develop effective markers for identifying the sex of *V. montana*, only one significantly sex-associated InDel located at Chr 2: 102 799 917-102 799 933 bp (keeping the lowest wald *p* value of 1.14e^−35^) was identified for primer designs (Supplementary Data Table S16). This InDel was homozygous in all 25 selected male individuals and heterozygous in 24 female individuals. The fragment length polymorphisms of the InDel marker in females and males were checked using the CE platform, with DNA samples obtained from 100 randomly selected *V. montana* individuals, comprising 50 females and 50 males. The results showed that the sex of *V. montana* could be identified by fragment length polymorphisms (Supplementary Data Fig. S20). Each male product (50 out of 50) showed only one peak at 253 bp (insertion of homozygote II) (Fig. 7d), whereas the majority of the female products (48 out of 50) showed two peaks at 249 bp and 253 bp (insertion/deletion of heterozygote ID) (Fig. 7e), displaying a 98% compliance rate. The sequences of the polymorphic fragments were further validated by Sanger sequencing (Fig. 7f). Finally, we demonstrated the InDel as a putative sex-linked InDel. Interestingly, the genotypes of GNN01 and SLS01, which were marked as females by field observation, were designated as homozygous (genotypic males) by both GATK4 and CE platforms based on the putative sex-linked InDel (Supplementary Data Table S17). Moreover, the putative sex-linked InDel is located in the second intron of *VmBASS4* (Fig. 6d).

## Discussion

The Euphorbiaceae family contains about 8000 species in 317 genera [16] with abundant economic and ornamental values. However, the genomes of only five species have been published to date [7, 17–20]. The assembly of the *V. montana* genome adds to the growth of the most recent genomes in the Euphorbiaceae family and provides a fundamental framework for studies of genomics, molecular breeding, and molecular biology in *V. montana.*

The COM clade is unique in Angiosperm Phylogeny Group IV (APG IV), and its phylogenetic position remains controversial [21, 22]. Plastid gene-based studies have shown that the Rosids are divided into two main clades: (1) Fabids, including the nitrogen-fixing clade (Fabales, Rosales, Fagales, and Cucurbitales), the COM clade, and Zygophyllales; (2) Malvids, including Brassicales, Malvales, Geraniales, etc. [23, 24]. Chloroplast and mitochondrial DNA are often inherited uniparentally and are widely affected by the proliferation of horizontal gene transfer (HGT) [25, 26]. Moreover, the mutation rate of nuclear genomes in land plants is ~10-fold and ~ 3-fold higher than that of mitochondrial and plastid genomes [27]. Thus, nuclear DNA may reflect the evolution of land plants more accurately than chloroplast and mitochondrial DNA. Inconsistency in species tree topologies is also influenced by inference methods. The traditional concatenation-based method assumes that all genes undergo the same evolutionary history, severely ignoring those inevitable evolutionary processes caused by the HGT, gene duplications and losses, as well as other problems that further complicate phylogenetic estimation, such as substitution saturation, long-branch attraction, and data loss [24]. The coalescent-based method is believed to be effective in solving these problems, but it remains to be discussed whether the concatenation-based or coalescent-based method is better when using large-scale datasets [27]. On the one hand, we inferred species phylogenetic relationships using identified single-copy genes in 21 chromosome-level angiosperm genomes, employing both concatenation-based and coalescent-based methods, respectively; on the other hand, we chose LCN OGs from 101 angiosperm genomes or transcriptomes to jointly construct the phylogenetic framework in this study. Both analyses support the conclusion that the COM clade is not monophyletic. The CM clade and Oxalidales are affiliated with malvids rather than fabids within rosids.

The analysis of the population structure indicated that *V. montana* individuals with the same ecological types are usually clustered together and share a similar genetic background (Fig. 5a–d). Based on the results of the best ancestry proportions, three subgroups could be clearly distinguished. The inconsistency in the estimation of LD, π ratios, and Tajima's D values of the three subgroups may be due to the complexity of the genetic backgrounds within the subgroups, suggesting that *V. montana* has a high degree of polymorphism, which is related to resource collection-based assisted migration with little to no artificial selection during modern cultivation. Several resistance genes were identified by selective sweep and genetic diversity analyses that underlie selection for environmental fitness during the evolution of *V. montana*.

*V. montana* is a dioecious economic woody plant, but there are no effective early sex identification approaches at present. In this study, a putative sex-linked InDel located at Chr 2: 102 799 917–102 799 933 bp in the *V. montana* genome was identified via GWAS, and this InDel polymorphic molecular marker can identify the sex of *V. montana* using the CE platform with a 98% compliance rate. The sex expression of *V. montana* male trees is susceptible to environmental influences that produce normally developing fruits [4]. Although GNN01 and SLS01 were identified as female through field observations, these trees were identified as male according to molecular characterizations. In our opinion, molecular characterizations are generally considered more accurate in plant sex identification. The InDel marker developed in this study perfectly identified the plant sex of *V. montana* trees. Moreover, the putative sex-linked InDels were found to be homozygous in male individuals and heterozygous in females, suggesting a possible ZW sex-determination system in *V. montana*. This is inconsistent with the proposed hypothesis of an XY sex system based on plant phenotypes in the field, indicating that determining plant sex based on phenotypes without molecular evidence is prone to error. A complete genome assembly is crucial for deciphering plant sex evolution and its important biological characteristics [28, 29]. For example, Liao et al. [30] generated a telomere-to-telomere genome for *Ficus hispida* and expanded the male-specific region from 2 Mb in the previously released genome [31] to a 7.2-Mb genomic region containing 51 newly identified genes. Due to the lack of a W-specific region in the male *V. montana* genome, a high-quality haplotype-solved female genome is needed to accurately identify the sex-determining region*.*

Several sex-specific genes in lineage-specific sex-determining regions have been reported to regulate sex determination in some dioecious flowering plants. Y-specific *AspTDF1* and *AspSOFF* are responsible for sex determination in garden asparagus, which influence anther development and female function, respectively [32, 33]. *VviFSEX* and *VviAPRT3*, located in the sex locus region on chromosome 2, are sex markers that allow the discrimination between male, female, and hermaphrodite *Vitis* plants [34]. In this study, we identified 84 sex-associated polymorphic loci, including 66 SNPs and 18 InDels, in three small regions on chromosome 2, some of which cover promoter or gene regions of four predicted protein-coding genes. Although *VmFRS5* and *VmBASS4* were barely expressed in flowers and *VmZC3H9* was nearly evenly expressed in both male and pistillate flowers, making their putative functions unclear, the location of the sex-linked InDel in the second intron of *VmBASS4* (Chr 2: 102 799 917–102 799 933 bp) suggests a possible role of *VmBASS4* in the sex determination in *V. montana. VmCET2* is expressed only during the early developmental stages of staminate flowers, suggesting a possible role in flower organ differentiation in staminate flowers of *V. montana*. The exact function of these putative sex-associated genes in *V. montana* needs to be further deciphered.

## Materials and methods

### Genome sequencing and assembly

#### Plant materials

A male tree of *V. montana* (Accession No. HXX37) was used for genome sequencing. All plant materials were conserved in the National Tung Tree Germplasm Conservation Repository located in Yongshun, Hunan Province, China (latitude 29°2′5″N, longitude 110°14′18″E).

#### Illumina sequencing

Young leaves were used to extract genomic DNA, and an Agilent 4200 Bioanalyzer (Agilent Technologies, Palo Alto, California) was used to assess the integrity of the DNA. The DNA was sequenced on the Illumina HiSeq Xten platform with 150-bp paired-end reads and 300 to 350 bp insert size, yielding a total of 62.8 Gb.

#### PacBio sequencing

About 22 μg of genomic DNA was sheared using g-Tubes (Covaris) and concentrated with AMPure PB magnetic beads for PacBio sequencing. The Pacific Biosciences SMRTbell™ template prep kit 1.0 was used to construct the libraries. Then the libraries were size-selected using a BluePippin™ system for molecules larger than 15 kb. Subsequently, primer annealing and SMRT bell template binding to polymerases were carried out using the DNA/Polymerase Binding Kit. Sequencing was performed at Wuhan Frasergen Bioinformatics Co., Ltd., China, for 10 hours using the PacBio Sequel platform.

#### Hi-C library construction and sequencing

Young leaves of *V. montana* (Accession No. HXX37) were utilized to construct Hi-C libraries, following the methodology outlined by Durand [35]. In brief, the leaves underwent a sequential set of steps that encompassed DNA fixation and crosslinking with 2% formaldehyde, overnight digestion of the DNA with *Mol* I overnight at 37°C; sticky end filling using Klenow polymerase and subsequent labeling with biotin-14-dCTP, ligation of the blunt-ended fragments with T4 DNA ligase at 16°C for 4 hours, purification of the ligation products, and shearing of the DNA into 300 to 500 bp fragments. Finally, the biotin-containing fragments were used to construct paired-end sequencing libraries. A total of 130.3 Gb of reads were generated on the Illumina NovaSeq 6000 system.

### Estimation of genome size

The estimation of the genome size was performed using a 17 bp K-mer distribution analysis with Jellyfish v2.0 [36] and a publicly available PERL script named estimated_genome_size.pl (https://github.com/josephryan/estimate_genome_size.pl).

### Genome assembly

For genome assembly, the PacBio CLR subreads were corrected and assembled using MECAT2 [37], resulting in 3487 contigs with an N50 length of 1.02 Mb. The initial assembly was then enhanced by using Hi-C data and was organized into 11 pseudo-chromosomes via the application of LACHESIS [38] software. Gap closure was achieved with the aid of LR_Gapcloser (https://github.com/CAFS-bioinformatics/LR_Gapcloser), while assembly errors were rectified using full-length transcripts through the Arrow tools integrated in SMRTlink v6.0. Finally, the assembly was polished with Pilon v1.22 (https://github.com/broadinstitute/pilon).

### Assessment of genome assembly

To evaluate the completeness of the final assemblies, 1614 conserved embryophyta groups (embryophyta_odb10) gathered from BUSCO (Benchmarking Universal Single-Copy Orthologs) were employed. Additionally, Illumina short reads were mapped onto the final assemblies utilizing BWA [39] and SAMtools, allowing us to evaluate read mapping rates. Notably, approximately 99.66% of the Illumina short reads were successfully mapped to the assembly, indicating a high level of completeness in the final assemblies based on Illumina data.

### Genome annotation

 

### RNA extraction and sequencing

Total RNA was extracted from a composite sample of seeds, leaves, roots, and flower buds. Subsequently, full-length transcriptome sequencing was conducted on the PacBio Sequel II platform, yielding 37.4 Gb of subreads with a N50 length of 1746 bp.

### Repeat annotation

The whole genome *de novo* TE annotation was generated by the EDTA package [40], showing that 78.58% of the male genome is occupied by transposable elements.

### Gene annotation

Using the MAKER pipeline [41], the gene structures were predicted, drawing upon evidence from *de novo*, homology-based, and transcriptome-based approaches. To minimize background noise resulting from frequently duplicated transposable elements (TEs), the genome assembly was masked using repetitive DNA sequences. Full-length transcriptome sequencing data underwent trimming with Trimmomatic [42] and was subsequently aligned to the genome assembly by Hisat2 [43]. Transcript ORF predictions were produced by TransDecoder (https://github.com/TransDecoder/TransDecoder). The gene models were trained using AUGUSTUS [44], based on the predicted gene structures. To facilitate further protein identification, GeneWise [45] was employed, utilizing homologous proteins from *V. fordii*, *J. curcas*, *R. communis*, *H. brasiliensis*, *M. esculenta*, *Arabidopsis thaliana*, and *Oryza sativa*. Incorporating all aforementioned evidence, a comprehensive set of gene predictions was generated via the MAKER pipeline. The BUSCO program [46], with embryophyta_odb10, served to evaluate the completeness of the gene annotation. Lastly, functional annotations for the genes were derived from public databases, including NCBI non-redundant protein sequence database (NR), SwissProt [47], Pfam [48], Gene Ontology [49], and KEGG [50].

### Non-coding RNA annotation

The transfer RNAs (tRNAs) were screened using tRNAscan-SE [51] with default parameters, while the ribosomal RNAs (rRNAs) were predicted by barrnap v0.9 (available at https://github.com/tseemann/barrnap). Additionally, the micro RNAs (miRNAs) and small nuclear RNAs (snRNAs) were annotated through the cmscan tool in the INFERNAL package (located at https://github.com/EddyRivasLab/infernal/) and were based on the RFAMSEQ10 database (accessible at http://ftp.ebi.ac.uk/pub/databases/Rfam/) using the recommended parameters.

### Genome evolution

#### The phylogenetic position of COM clade (including Celastrales, Oxalidales, and Malpighiales)

The single-copy gene families of 21 species, each possessing a sequence length exceeding 100 bp (Supplementary Data Table S18), were identified utilizing the OrthoMCL [52] results. Leveraging Maximum Likelihood (ML) methods, concatenated and coalescent trees were constructed from both amino acid and nucleotide sequences. For concatenated trees, the amino acid and nucleotide sequences from 141 identified single-copy families were assembled to create respective supergene datasets. As for coalescent trees, orthologous genes from the 141 single-copy families within the selected species were grouped into orthologue datasets. Muscle v3.8 was employed to align the protein sequences, which were subsequently converted into codon alignments using PAL2NAL [53]. trimAl [54] was then utilized to refine the alignment results, applying the ‘-automated1’ parameter. The ML trees were built with RAxML [55], specifying ‘-m PROTGAMMAJTT’ for amino acid sequences and ‘-m GTRGAMMA’ for nucleotide sequences. To finalize the coalescent trees, the phylogenetic data from the orthologue datasets were combined using Astral (https://github.com/sffjunkie/astral). Furthermore, to estimate the evolutionary timescale of the 21 species, the MCMCTree tool from the PAML package [56] was applied, based on the concatenated tree's topological structure with nucleotide sequences. Six divergence times retrieved from the TimeTree database [57] (September 2020) served as calibration points (Supplementary Data Table S19).

Utilizing reported methodologies [23], phylogenetic trees encompassing nuclear genes from 101 species, comprising 83 genomic and 18 transcriptomic datasets, were constructed (Supplementary Data Table S20). Initially, orthologous LCN genes of the selected species were identified by comparing their genes against a comprehensive LCN gene set containing 1167 genes obtained from Waterlily Pond (http://waterlily.eplant.org) [23]. Subsequently, the phylogenetic tree reconstruction involved a multi-step process involving carefully selected orthologous groups (OGs): (1) a total of 1087 OGs were extracted, encompassing orthologous LCN genes present in over 50% of the selected species; (2) of these 1087 OGs, 970 were further refined based on a minimum sequence length of 800 bp and a species coverage of 80%; (3) the selection was narrowed down to 748 OGs, which possessed a minimum sequence length of 1000 bp and a species coverage of 90%; (4) from the 748 OGs, 627 were retained, specifically those containing six basal species: *Gnetum montanum*, *Picea abies*, *Ginkgo biloba*, *Amborella trichopoda*, *Nymphaea colorata*, and *Austrobaileya scandens*; (5) finally, 125 OGs with a species coverage of 100% were chosen for the reconstruction of coalescent trees using coding sequences, following the aforementioned methodology. Additionally, molecular dating was conducted, leveraging age calibration data provided by TimeTree (September 2020) as a reference (Supplementary Data Table S21).

### Whole-genome duplication

To identify whole genome duplication events, the JCVI pipeline [58] was employed to determine the syntenic blocks within *V. montana*, as well as between *V. montana* and *V. fordii*, *J. curcas*, *H. brasiliensis*, *M. esculenta,* and *R. communis,* and *V. vinifera*. Given the significant repeat content in the *V. montana* genome, the parameter ‘—cscore = 0.99’ was applied to eliminate short syntenic blocks potentially resulting from transposon elements. Additionally, comparative genomic analyses were conducted between *V. montana*, other Euphorbiaceae species, and *V. vinifera* using the pre-γ AEK sequences [59].

Utilizing the codeml method from the PAML package, we calculated the non-synonymous (*K_a_*) and synonymous (*K_s_*) nucleotide substitutions, along with the *ω* ratio (*K_a_*/*K_s_*), for the syntenic block genes. The peak values of the *K_s_* distribution were precisely identified using the Findpeaks function in the PRACMA package (available at https://github.com/cran/pracma). To assess the timing of the WGT-*γ* event in *V. montana*, we converted the calculated peak *K_s_* values of its syntenic block genes into divergence time, applying the formula T = *K_s_*/2r, and using an r-value of 6.5 × 10^−9^ synonymous substitutions per site per year for eudicots. We dated the *V. montana* WGT-*γ* (*K_s_* = 1.513) to approximately 116.4 Mya. Following the same methodology, we also estimated the divergence time between *V. montana* and other species.

### Gene expression analysis

The transcriptome data for flowers of *V. montana* at various developmental stages were obtained from GSA: CRA006657, and the corresponding FPKM values were calculated using the methodology outlined in Li's work [4].

### Population evolution and genetic diversity

The fresh tender leaves of 178 *V. montana* trees were collected across 10 distinct distribution areas: (I) SLS in Sichuan province, (II) YKM in Yunnan Province, (III) GNN in Guangxi Province, (IV) HXX and (V) HCZ in Hunan Province, (VI) HWH in Hubei Province, (VII) JYC in Jiangxi Province, (VIII) ZLS and (IX) ZHZ in Zhejiang Province, and (X) GSG in Guangdong Province (Supplementary Data Table S22). Among these 178 trees, 88 were males and 90 were females, with sex determination based on the observation of inflorescence characteristics and fruit-bearing patterns.

DNA was extracted from leaves dried with silica gel using DNeasy Plant Pro Kit (Qiagen), followed by whole genome resequencing on the Illumina Novaseq 6000 platform with an average sequencing depth of 12X. The resulting reads were aligned to the reference genome using BWA [39] with default parameters. Variant identification was carried out using Sentieon genomics software (Version 2017) [60]. High-quality SNPs and InDels were generated using the Genome Analysis ToolKit (GATK4) [61] and VCFtools [62]. A neighbor-joining phylogenetic tree with 1000 bootstrap replicates was constructed based on SNPs within single-copy gene regions using TreeBeST (https://treesoft.sourceforge.net). These SNPs were also used in a PCA analysis performed by Plink (https://github.com/chrchang/plink-ng). Ancestral population structure was estimated employing ADMIXTURE [63] software with 1000 bootstraps replicates. Linkage disequilibrium was estimated using PopLDdecay [64] software. Nucleotide diversity (π) ratios, Tajima’s D values, and fixation index (*Fst*) were calculated using VCFtools [62].

### Genome-wide association analysis

SNPs and InDels from 25 females and 25 males (Supplementary Data Table S13) were extracted for genome-wide association analysis. To assess the statistical association between genetic polymorphisms and sex in *V. montana*, the GEMMA software [65], employing a standard linear mixed model, was utilized. The validity of this model was verified using QQ-plots comparing observed and expected *p* values (Supplementary Data Fig. S21). Subsequently, significant sex-associated polymorphisms were identified using the Genetic Type I error calculator [66], based on the *p*-values derived from the Wald test executed by GEMMA [65]. LD maps were generated by LDBlockShow [67].

### InDel validation through Sanger sequencing

Sequences spanning 300 base pairs upstream and downstream of the identified significant sex-associated InDel were extracted, and primers for amplifying the region was designed using SnapGene (Supplementary Data Table S16). 6-FAM-tagged fluorescent dye primers were synthesized. Individual DNA fragments extracted from 100 randomly selected samples (50 females and 50 males) (Supplementary Data Table S22) were isolated using the capillary electrophoresis (CE) platform provided by Sangon Biotech Co., Ltd, Shanghai, China. The fragment sizes were determined through data analysis software (GeneMapper), employing 500 LIZ (GeneScan™) as the size standard. To verify the polymorphic fragments, conventional PCR and Sanger sequencing were performed on representative samples HXX09 (female) and HXX60 (male).

## Supplementary Material

Web_Material_uhae141

## Data Availability

The PacBio sequencing data, Hi-C data, ISO-seq data, and resequencing short reads have been deposited in the Genome Sequence Archive (Genomics, Proteomics & Bioinformatics 2021) in the National Genomics Data Center (Nucleic Acids Res 2022), China National Center for Bioinformation / Beijing Institute of Genomics, Chinese Academy of Sciences (GSA: CRA007017 and CRA009450) that are publicly accessible at https://ngdc.cncb.ac.cn/gsa.
