# Supplementary material for: Genome assembly and resequencing shed light on evolution, population selection, and sex identification in Vernicia montana
Source: Hortic Res. 2024 May 18;11(7):uhae141. doi: 10.1093/hr/uhae141 (PMC11233859; doi:10.1093/hr/uhae141)
Supplement: Web_Material_uhae141 [file web_material_uhae141.zip › Supplementary_figures.docx]

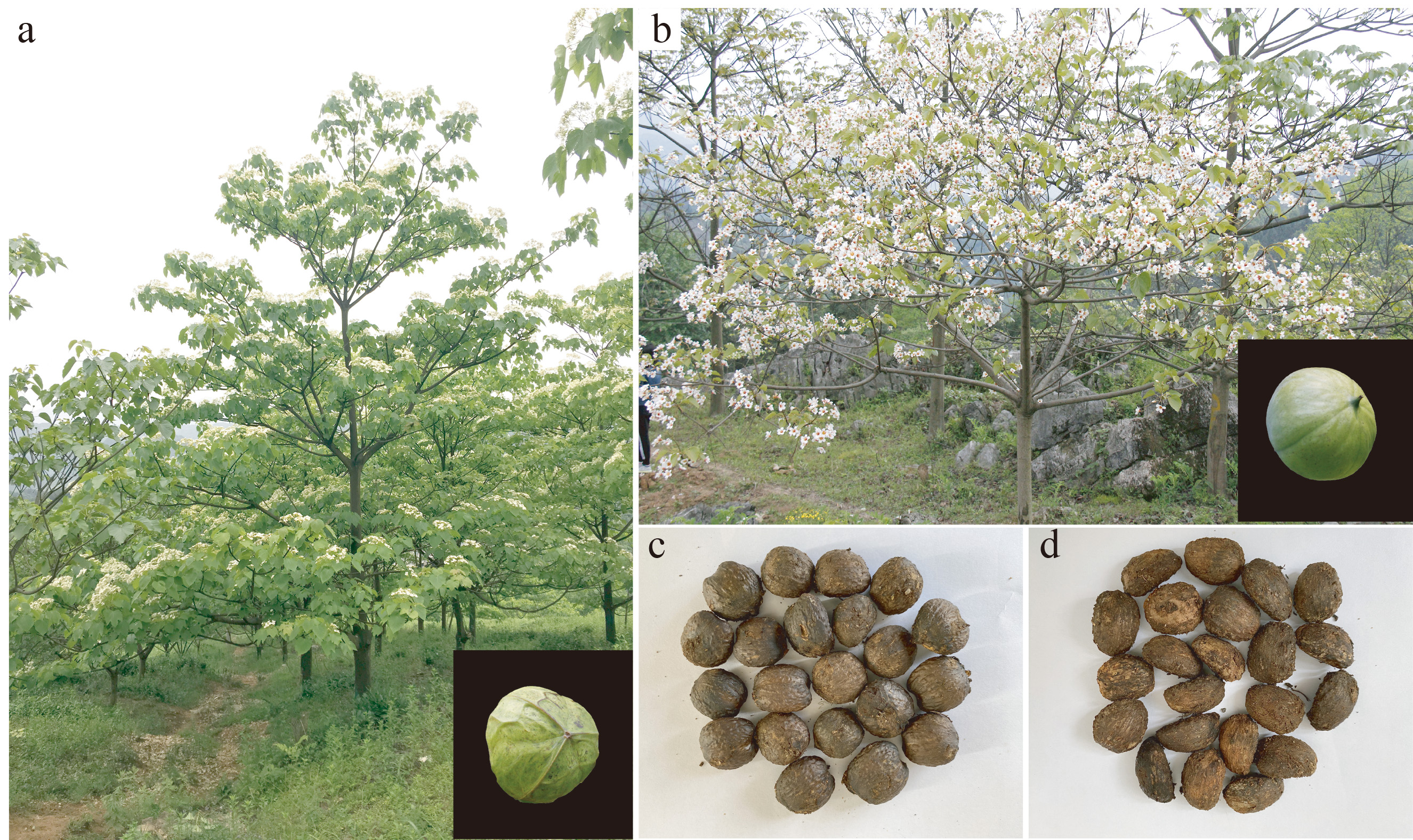


**Figure S1.** The two species of tung oil trees. (a) A flowering male *Vernicia montana* and a fruit. (b) A flowering monoecious *Vernicia fordii* and a fruit. (c) and (d) are seeds of *V. montana* and *V. fordii*, respectively.


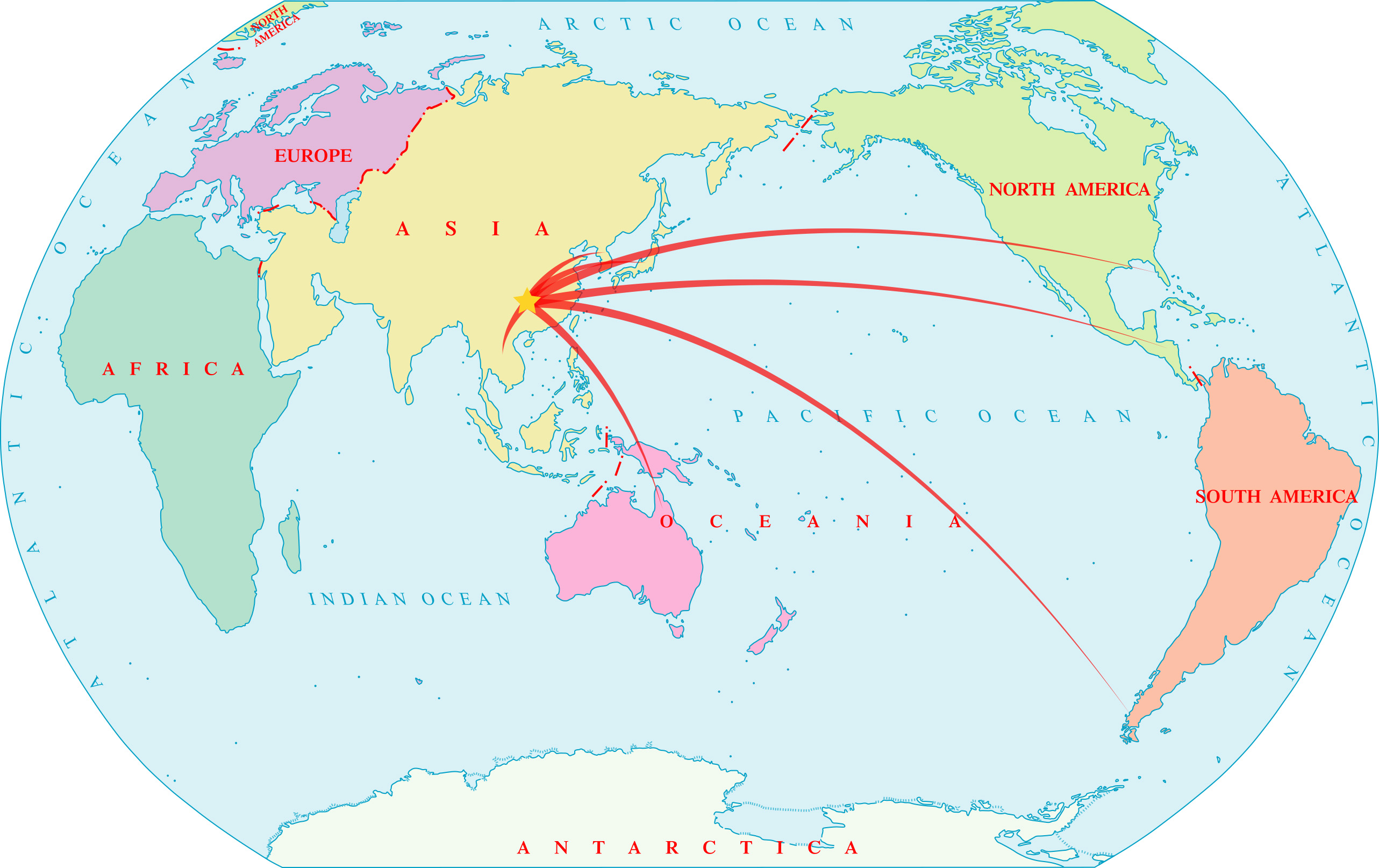


**Figure S2.** Worldwide distribution of *V. montana*. Dark grey blocks represent distribution areas of *V. montana.*


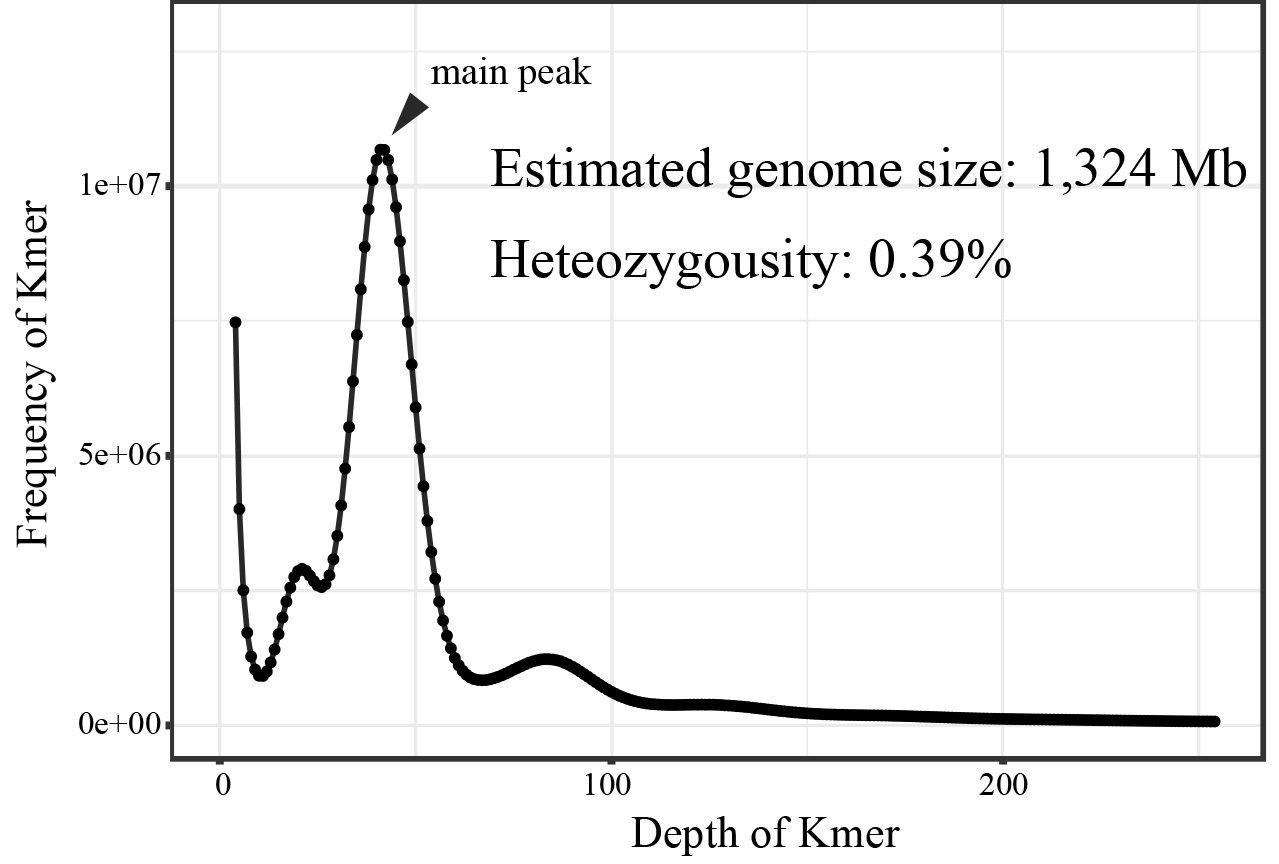


**Figure S3.** The *K*-mer (17-mer) distribution and estimation of genome sizes and heterozygosity rates of *V. montana*.


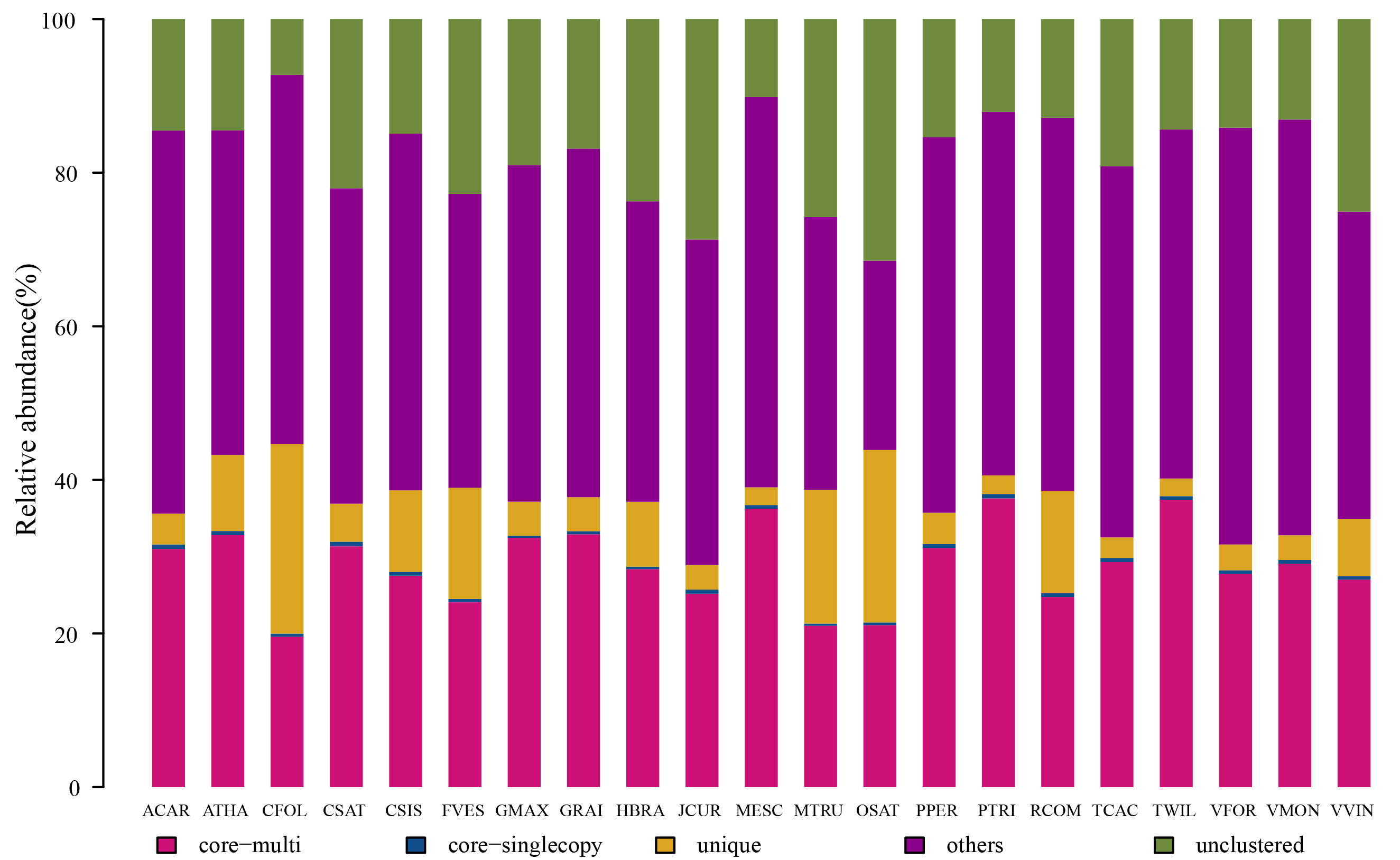


**Figure S4.** Gene family clustering of *V. montana* and 20 other species.


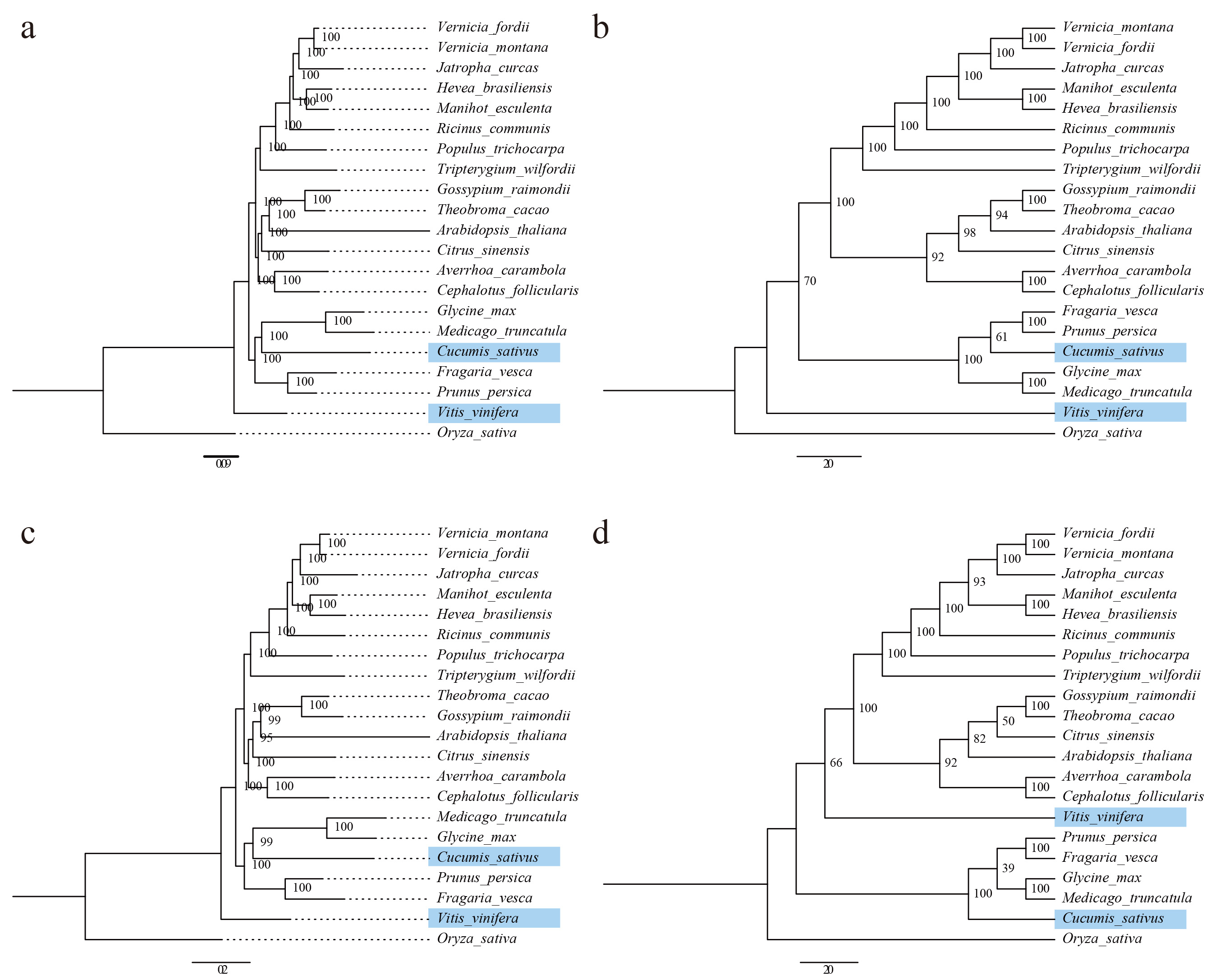


**Figure S5.** Phylogenetic trees constructed by multiple methods. (a) Concatenated tree constructed using coding sequences; (b) Coalescent tree constructed using coding sequences; (c) Concatenated tree constructed using protein sequences; (d) Coalescent tree constructed using protein sequences. Note: The phylogenetic positions of species marked blue were inconsistent in different trees.


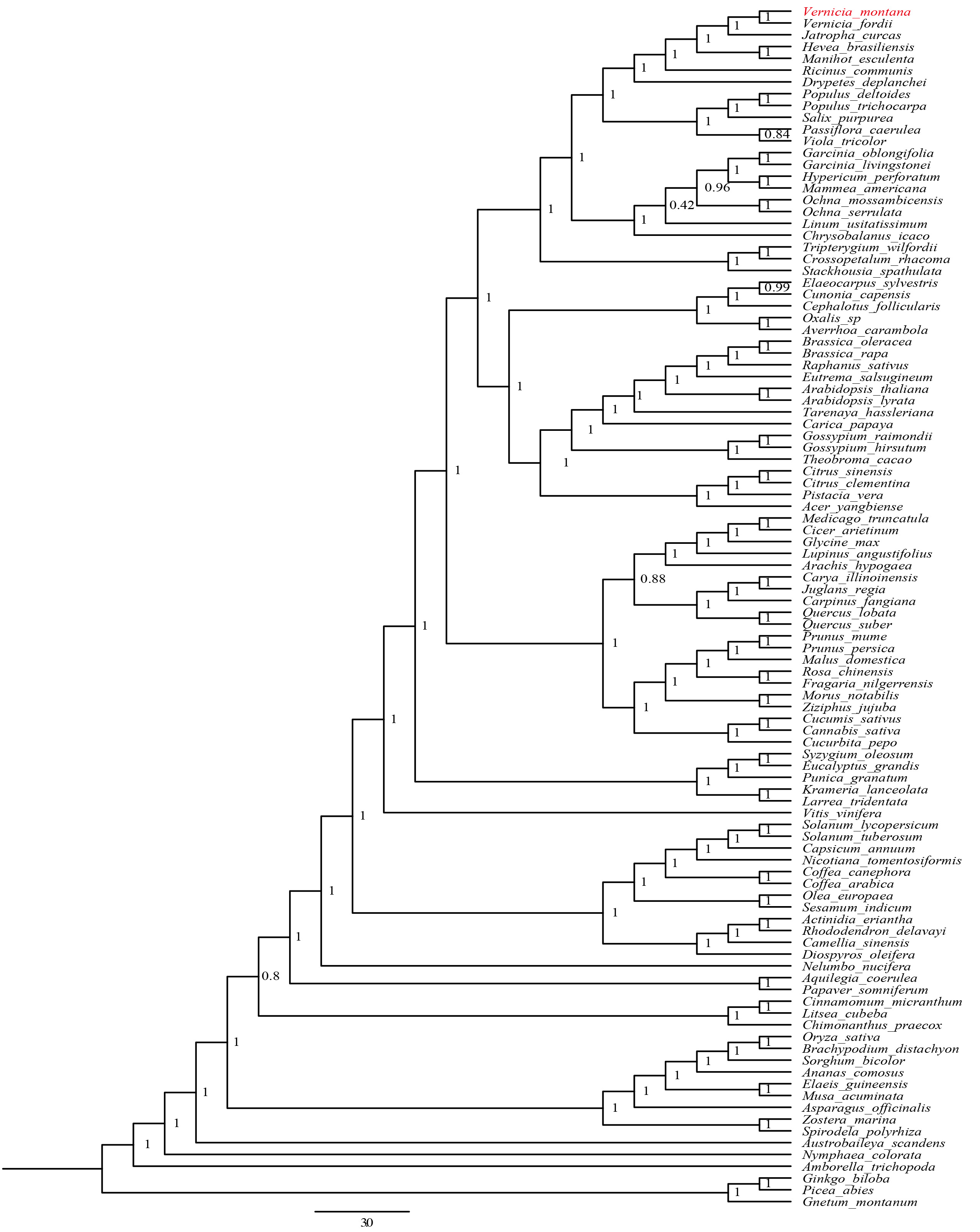


**Figure S6.** The concatenated tree of coding sequences of 1,087 orthologous groups in 101 angiosperms.


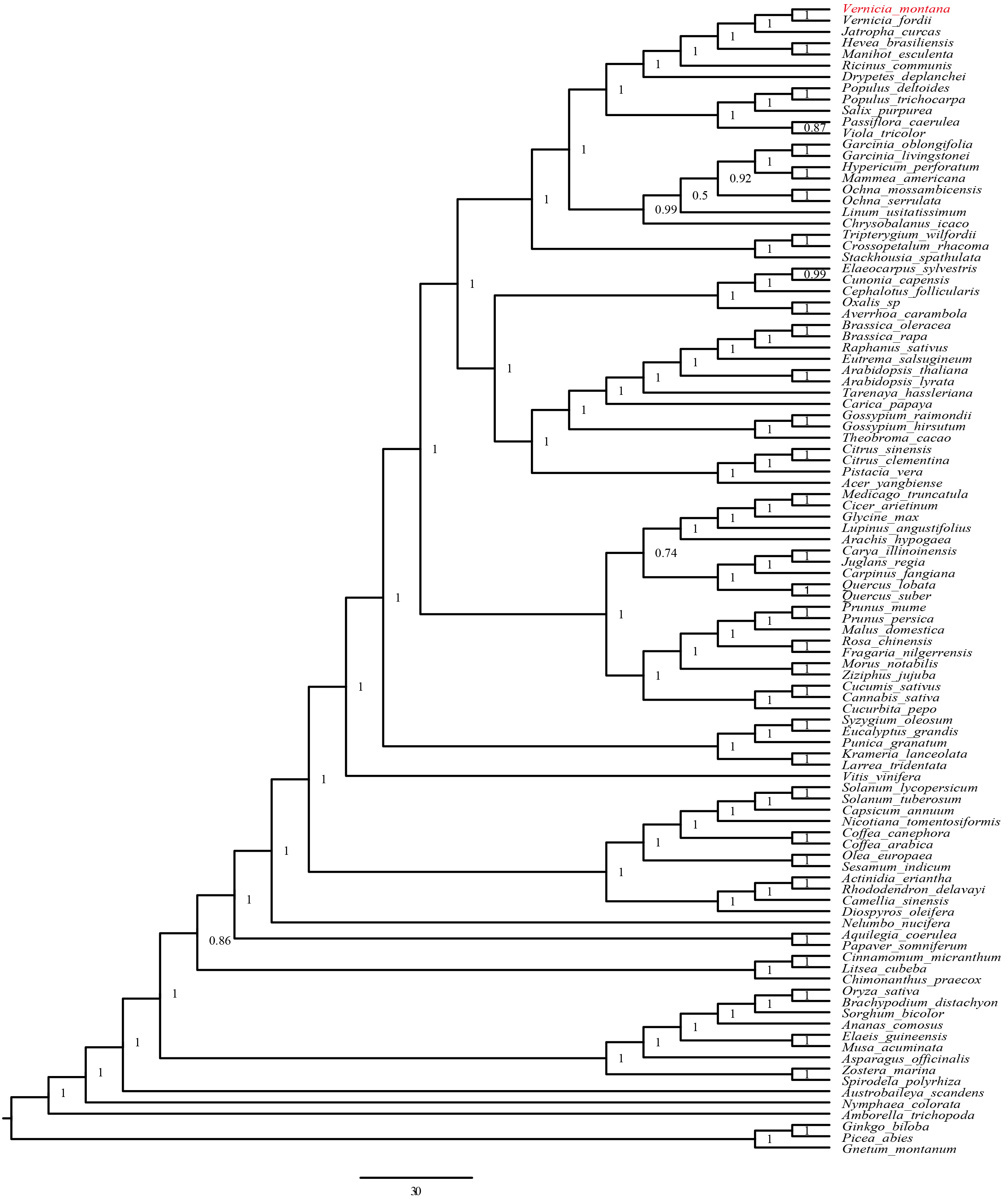


**Figure S7.** The concatenated tree of coding sequences of 970 orthologous groups in 101 angiosperms.


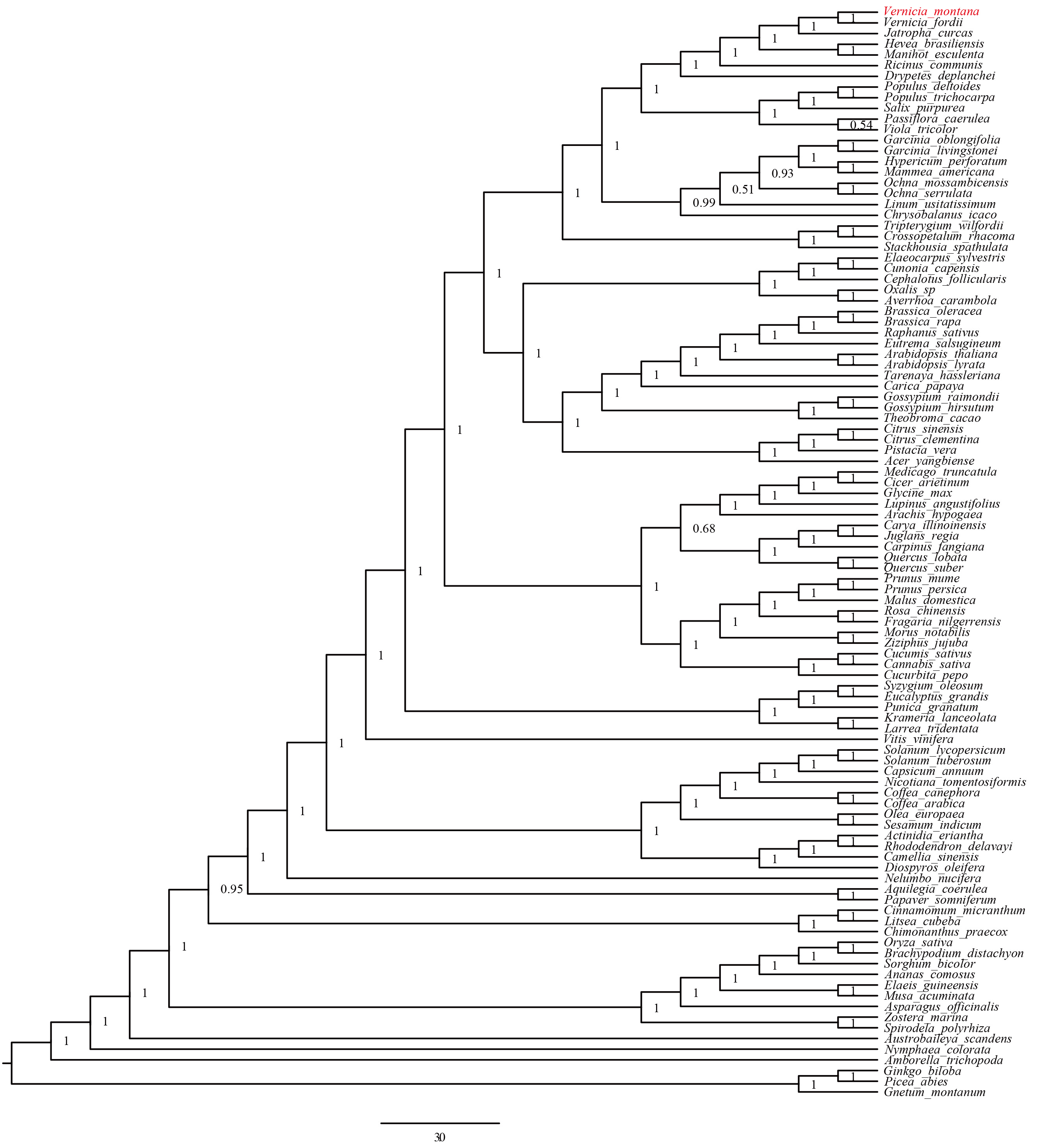


**Figure S8.** The concatenated tree of coding sequences of 748 orthologous groups in 101 angiosperms.


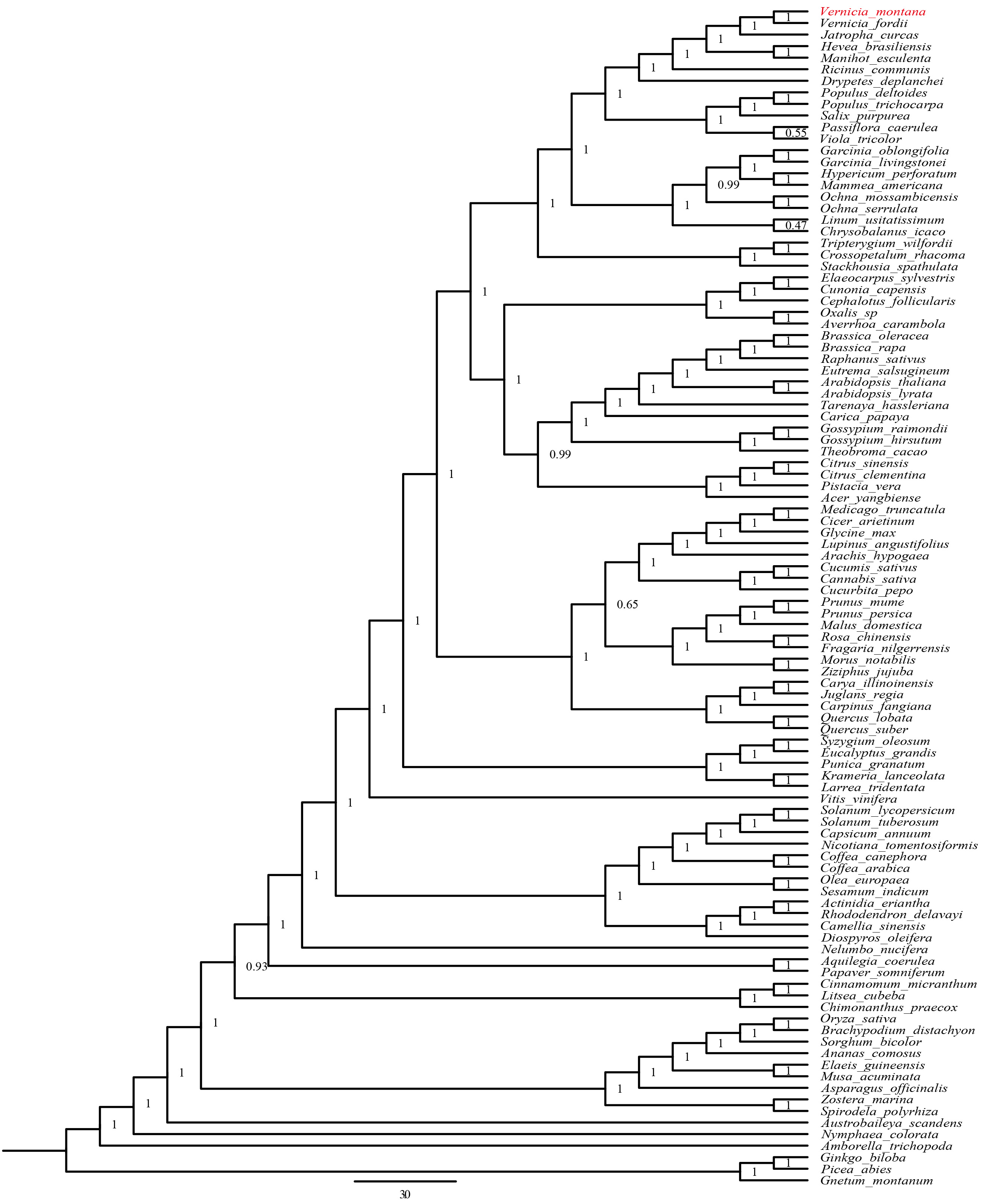


**Figure S9.** The concatenated tree of coding sequences of 627 orthologous groups in 101 angiosperms.


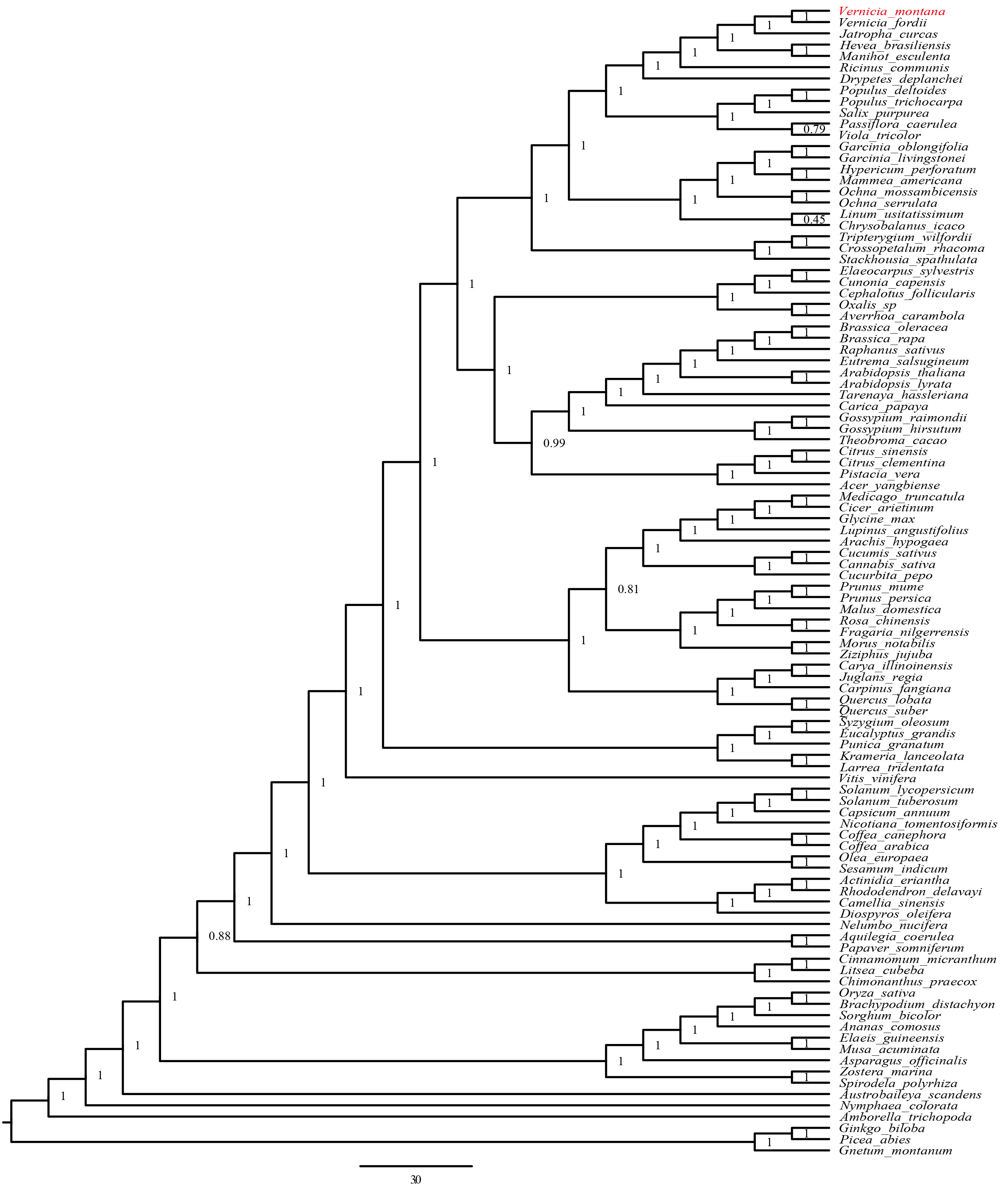


**Figure S10.** The concatenated tree of coding sequences of 125 orthologous groups in 101 angiosperms.


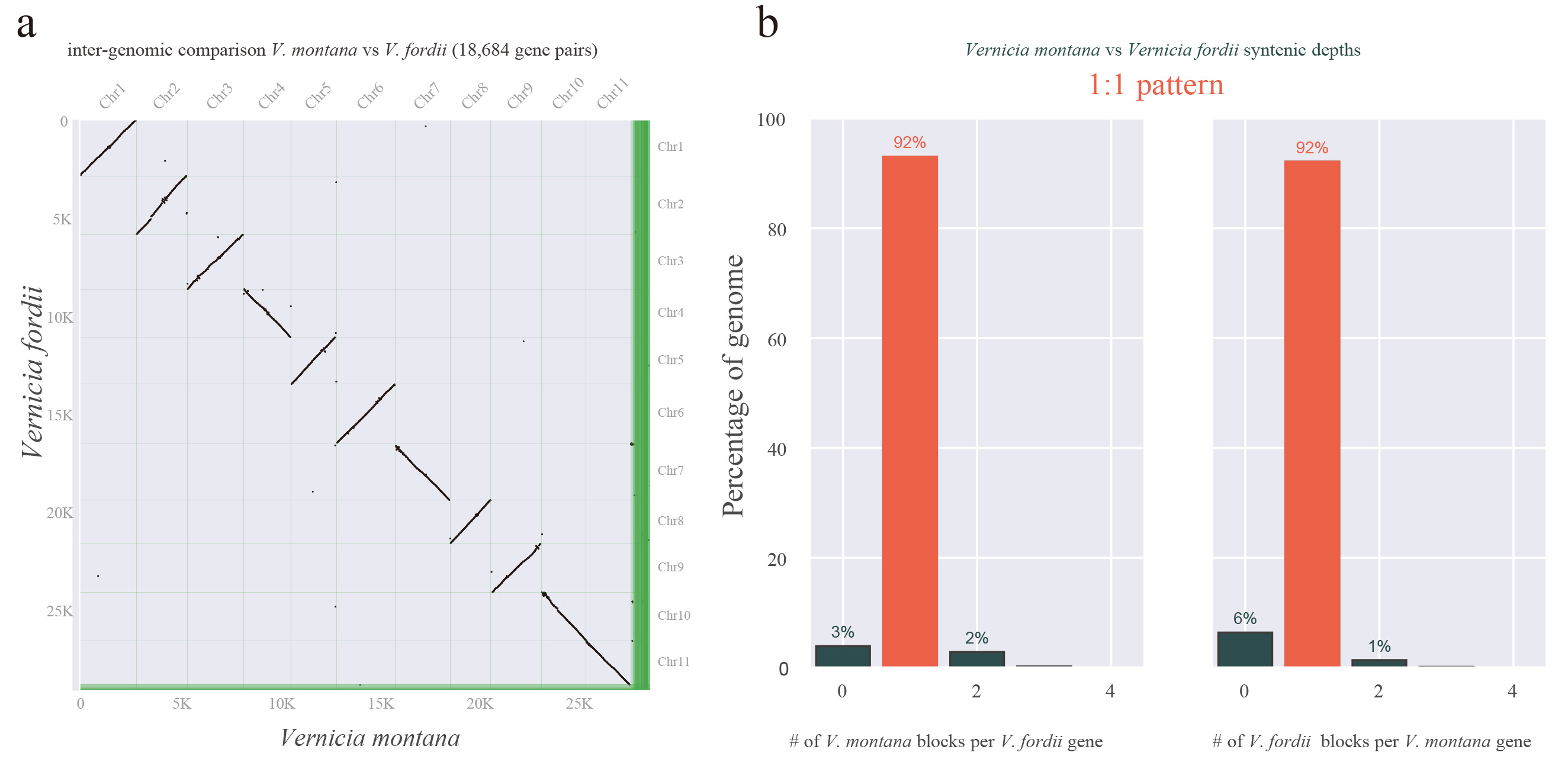


**Figure S11.** Syntenic dot plots (a) and syntenic depths (b) between *V. montana* and *V. fordii.*


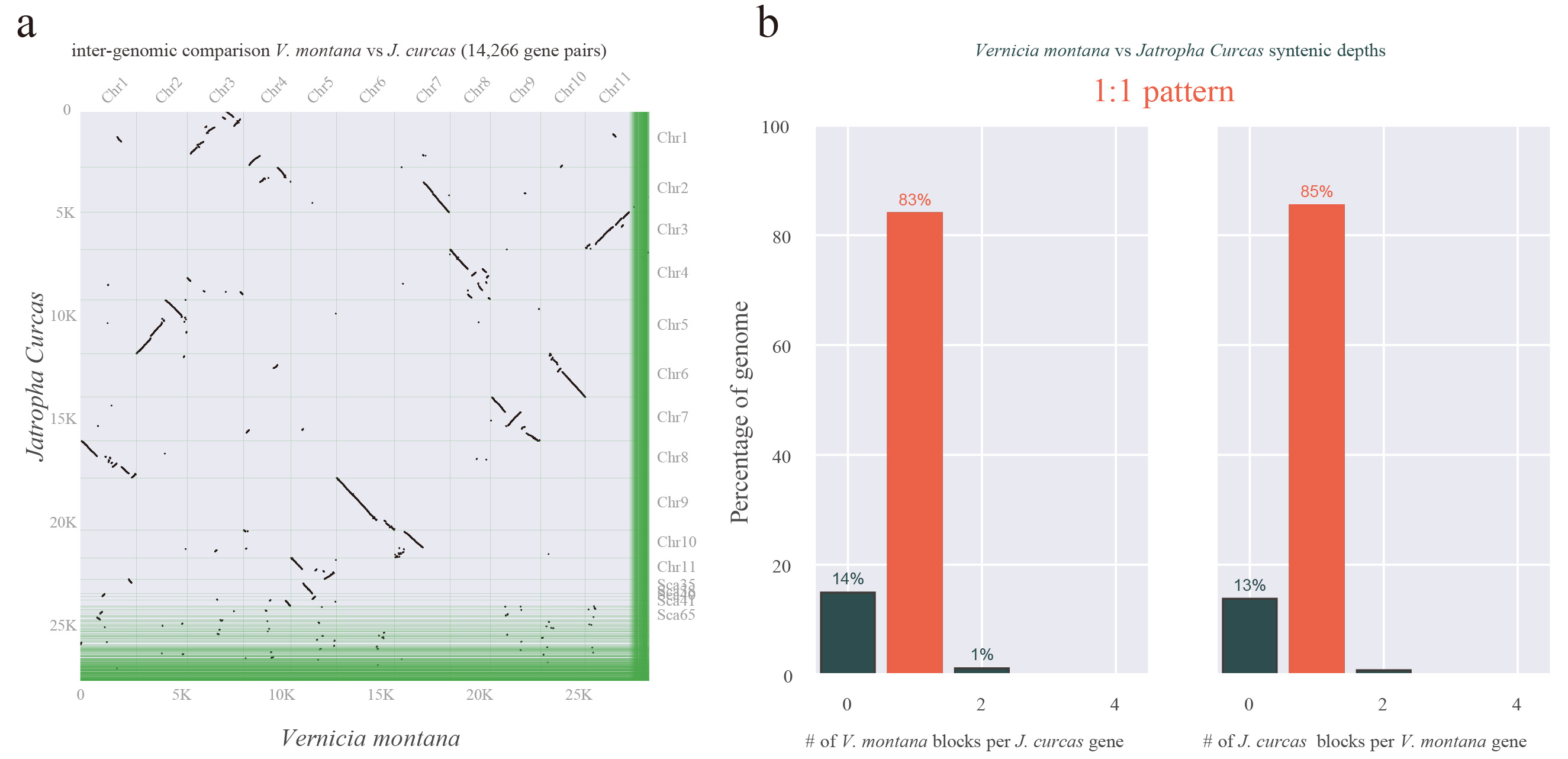


**Figure S12.** Syntenic dot plots (a) and syntenic depths (b) between *V. montana* and *J. curcas.*


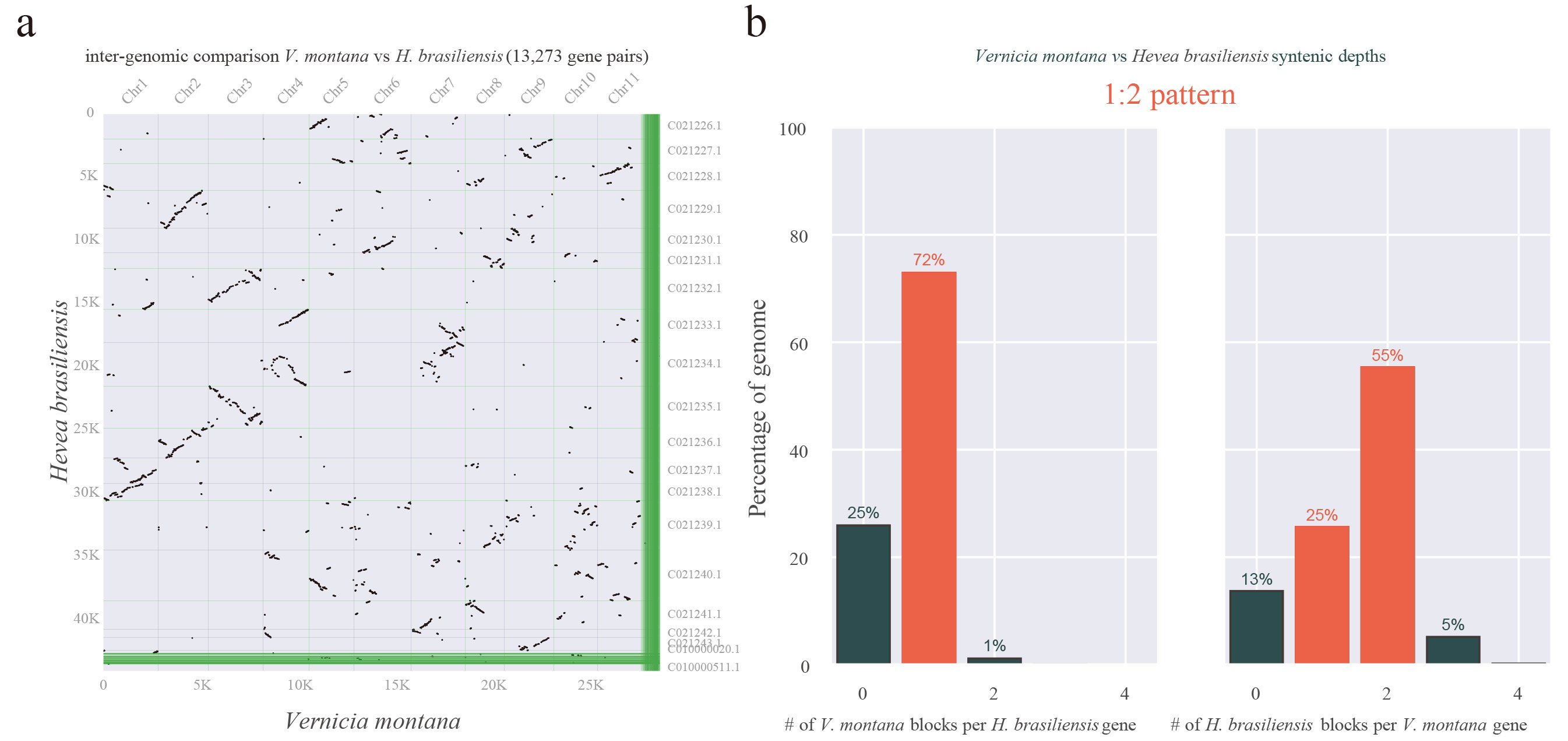


**Figure S13.** Syntenic dot plots (a) and syntenic depths (b) between *V. montana* and *H.brasiliensis.*


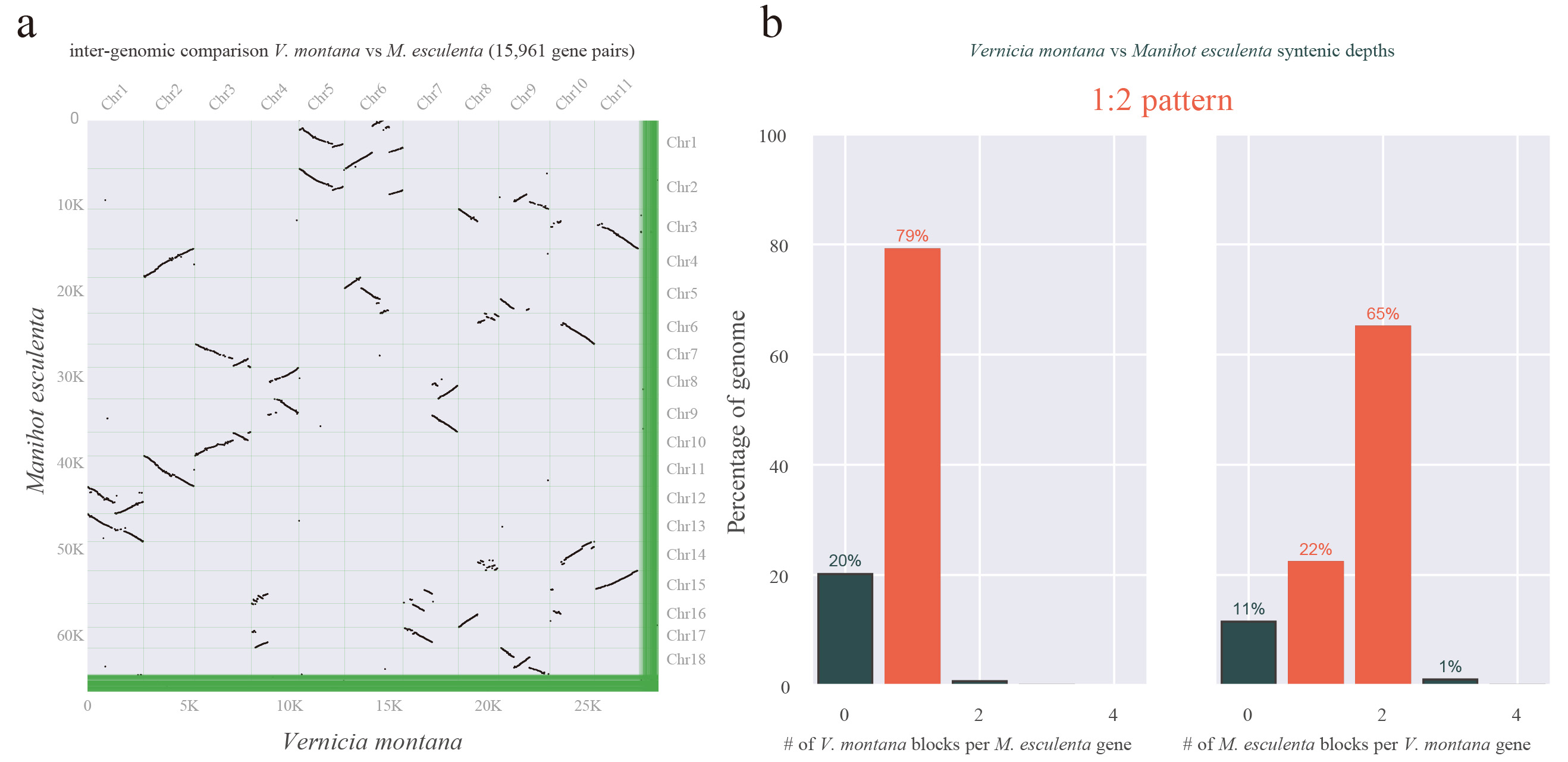


**Figure S14.** Syntenic dot plots (a) and syntenic depths (b) between *V. montana* and *M. esculenta.*


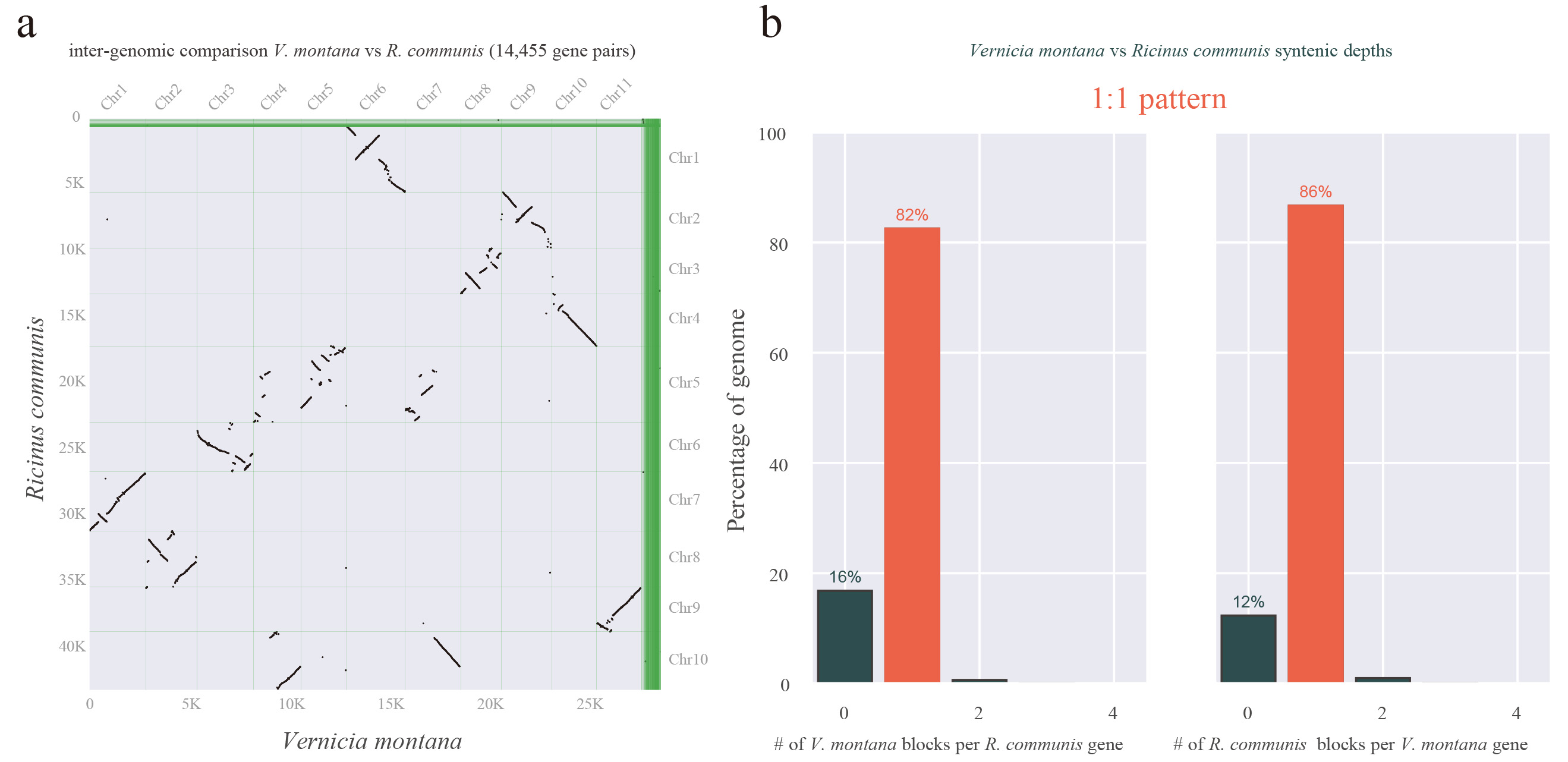


**Figure S15.** Syntenic dot plots (a) and syntenic depths (b) between *V. montana* and *R. communis.*


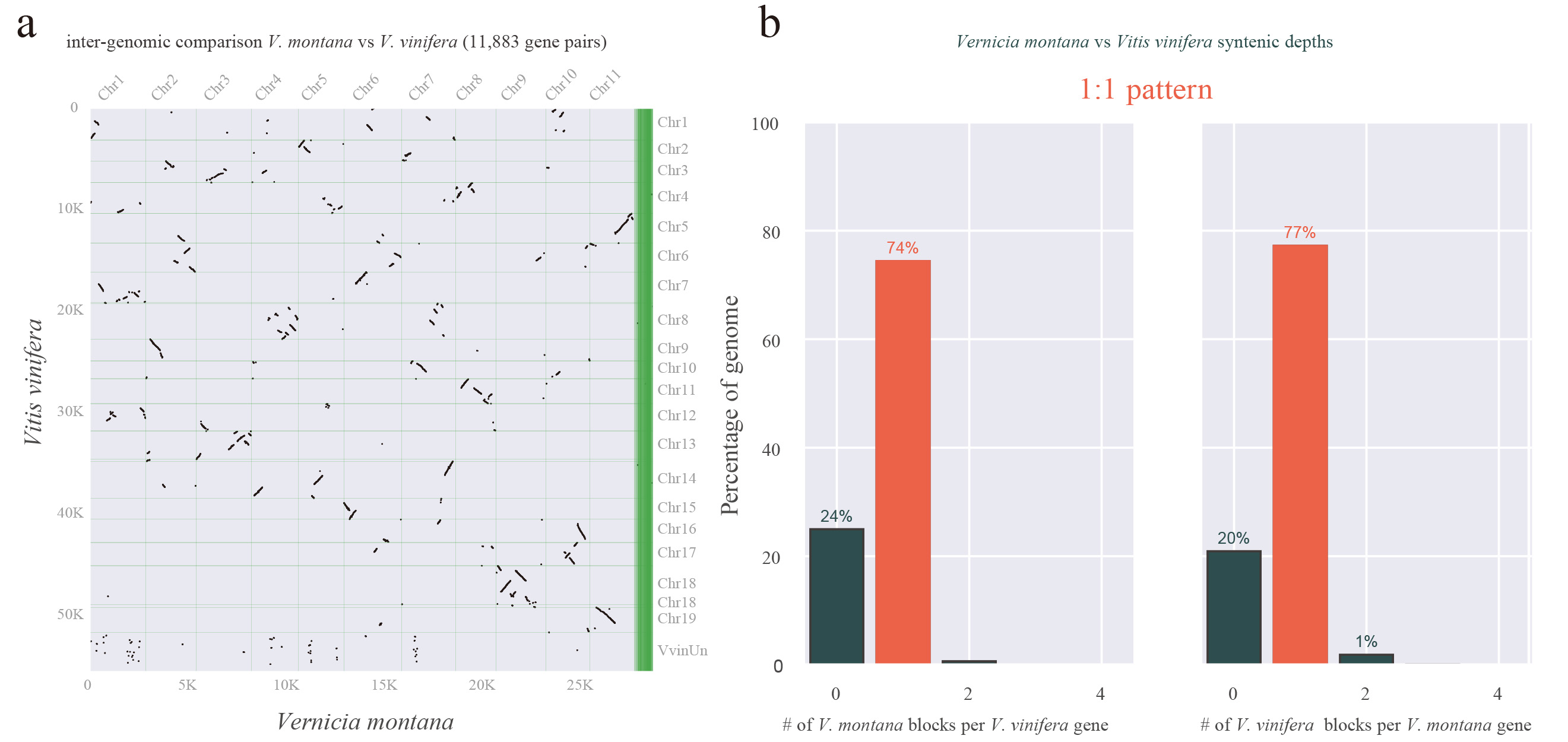


**Figure S16.** Syntenic dot plots (a) and syntenic depths (b) between *V. montana* and *V. vinifera.*


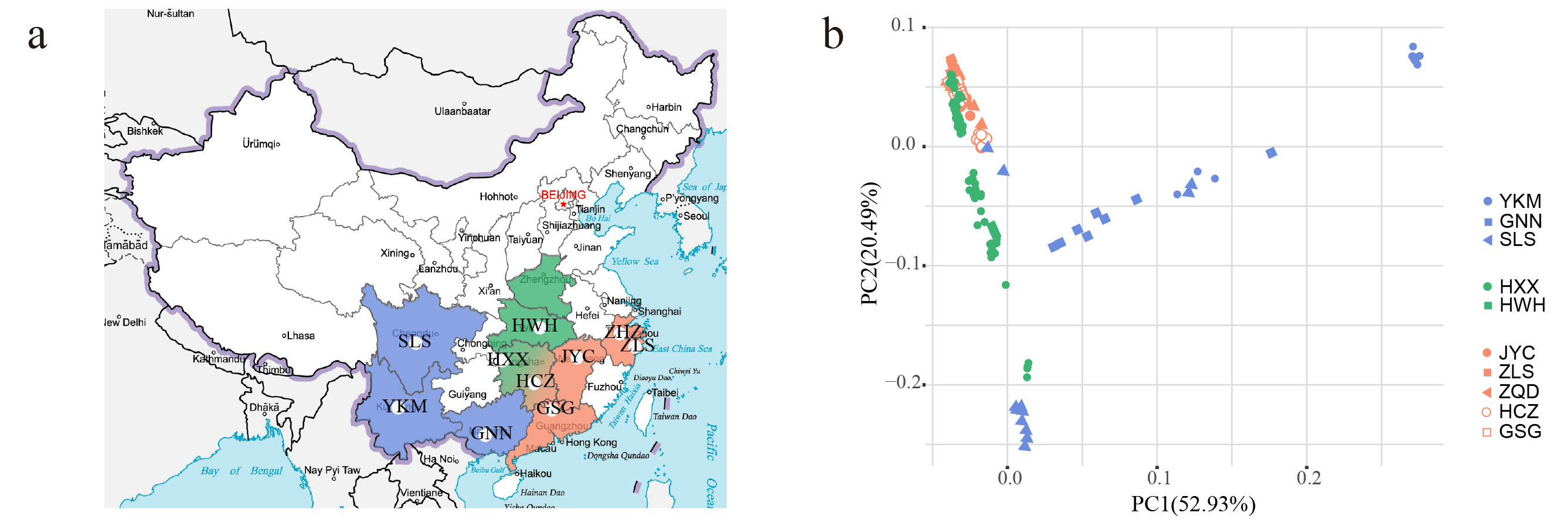


**Figure S17.** Sample collection site (a) and principal component analysis (b) for 178 *V. montana* individuals from 10 different ecological populations.


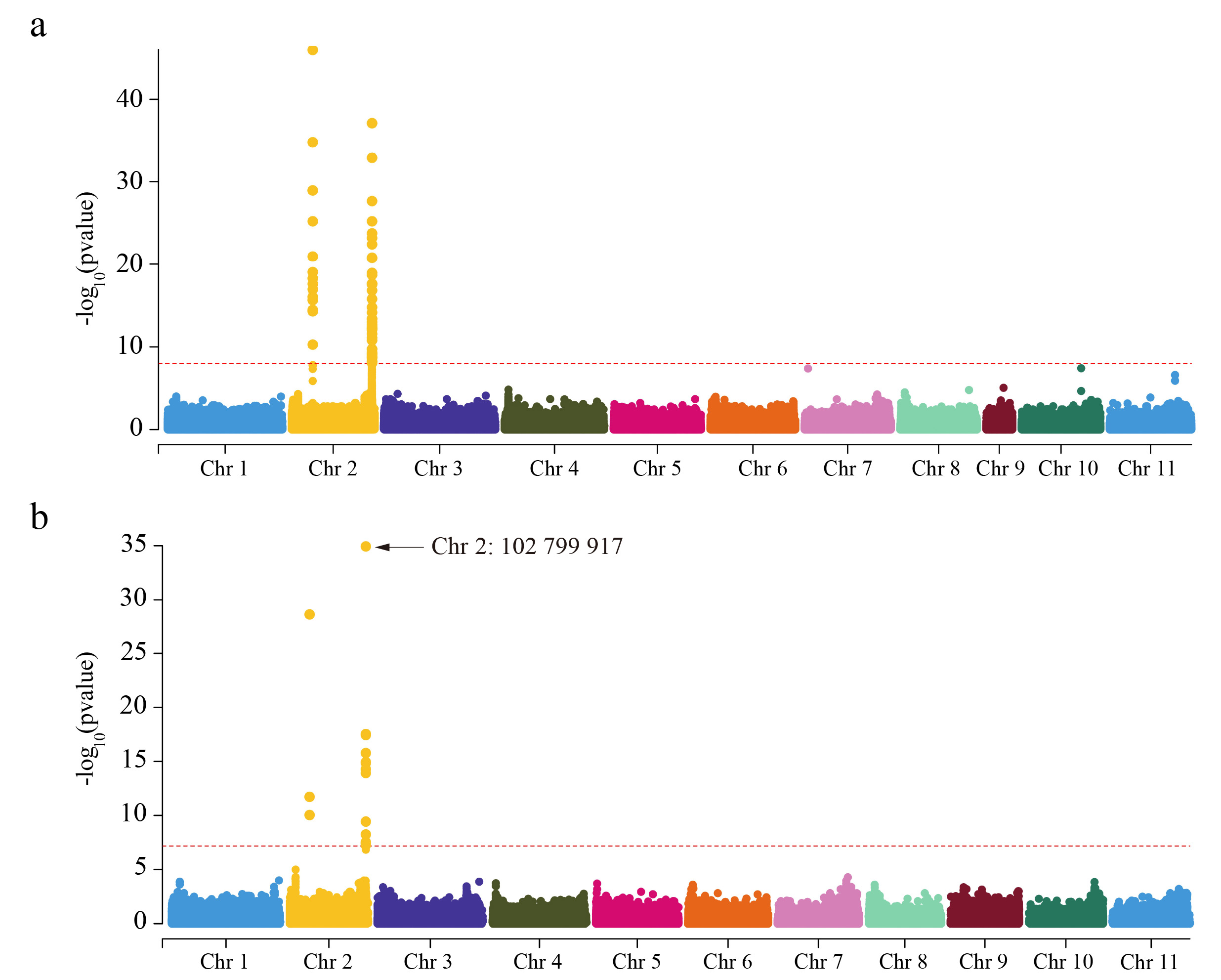


**Figure S18.** Manhattan plots of genome-wide association studies (GWAS) results based on SNPs (a) and InDels (b).


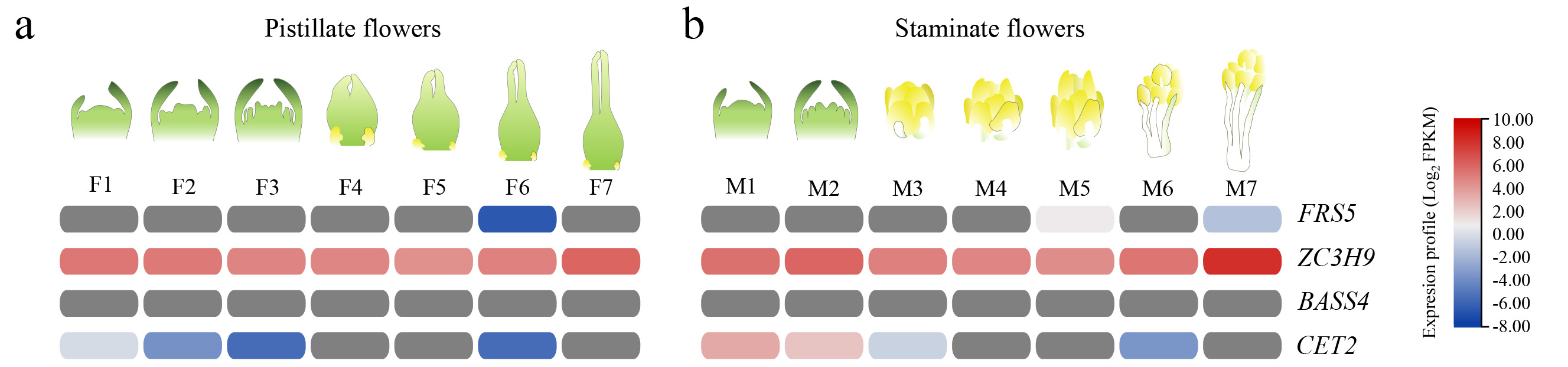


**Figure S19.** Expression profiles of genes with at least one variant identified by GWAS.


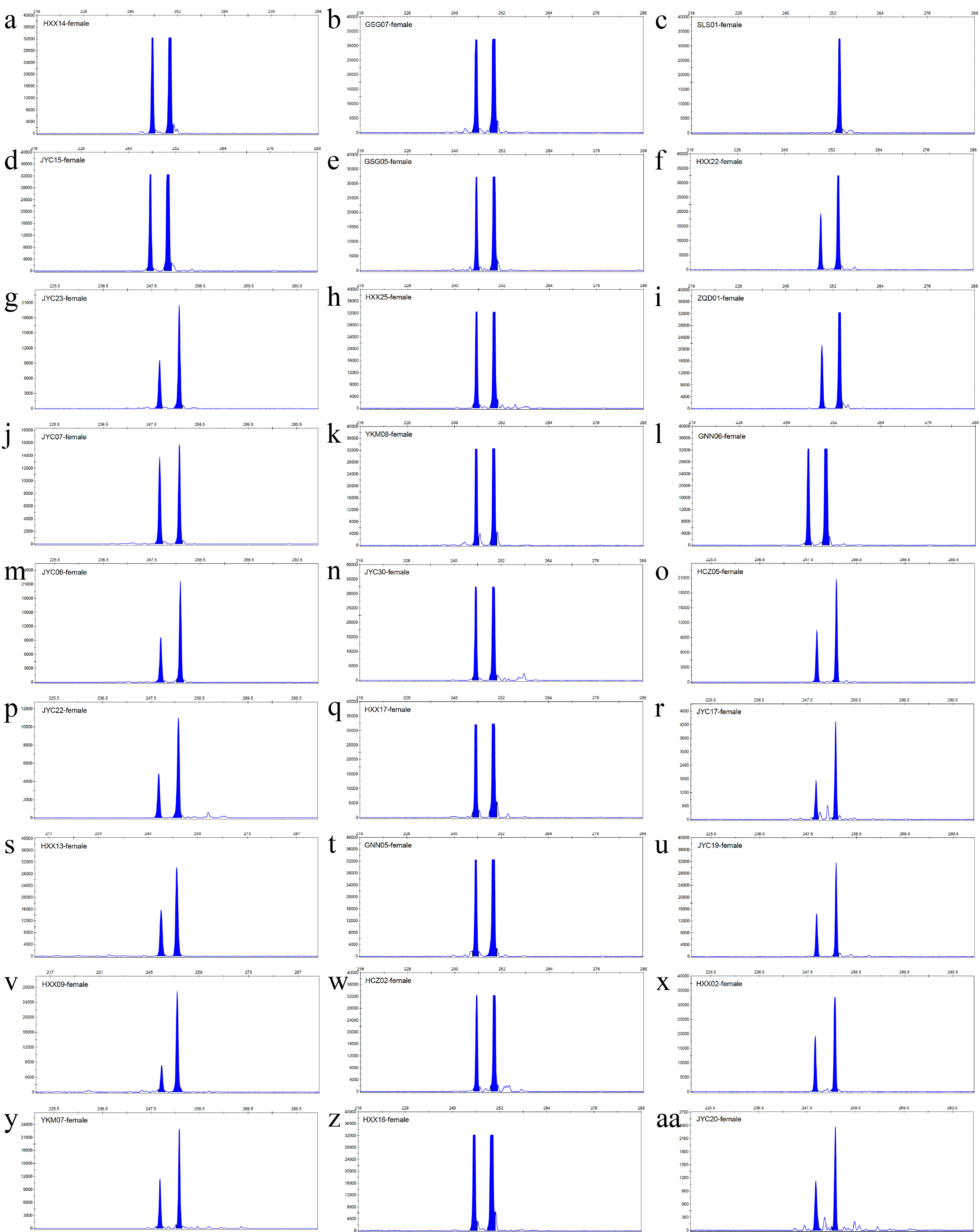


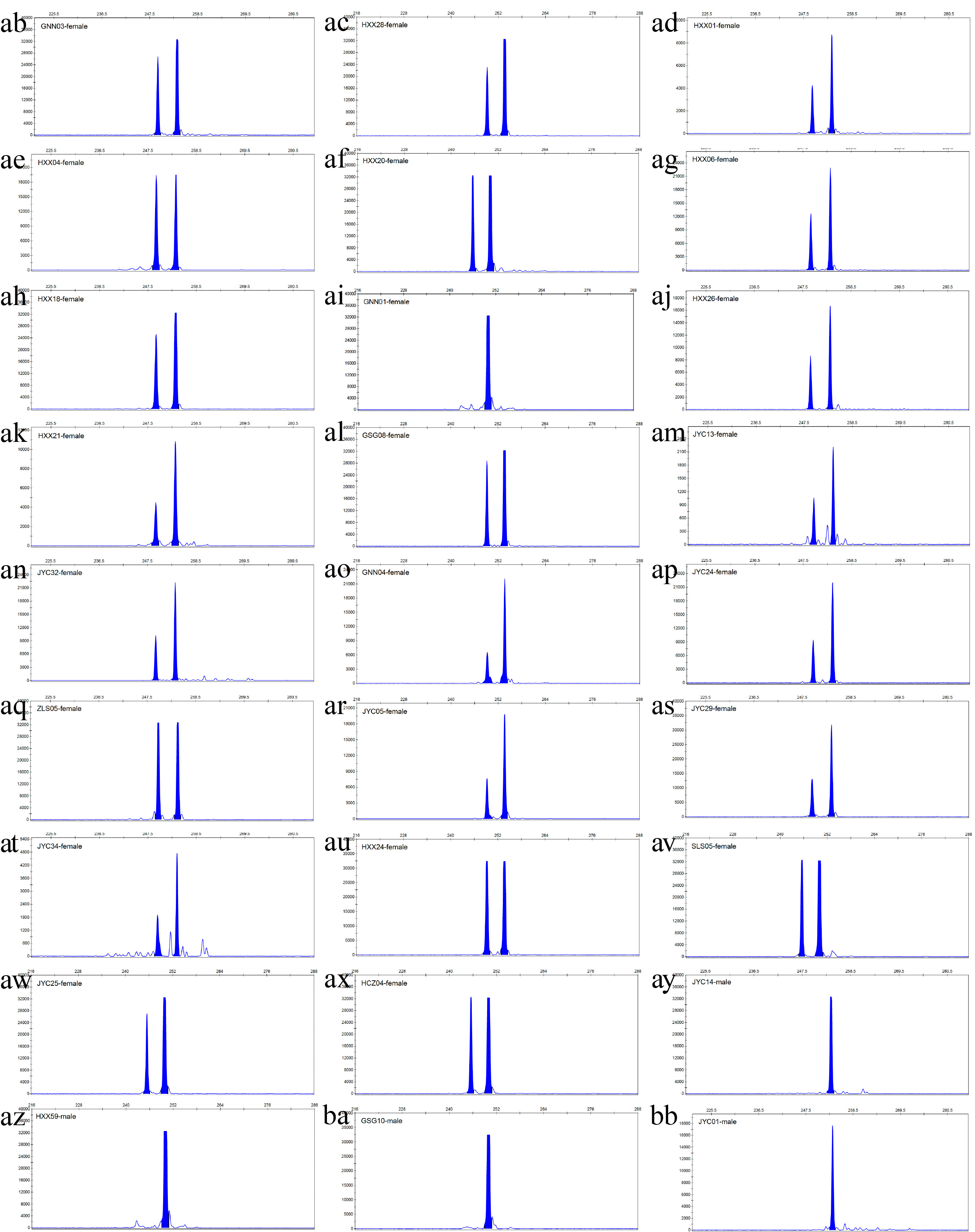

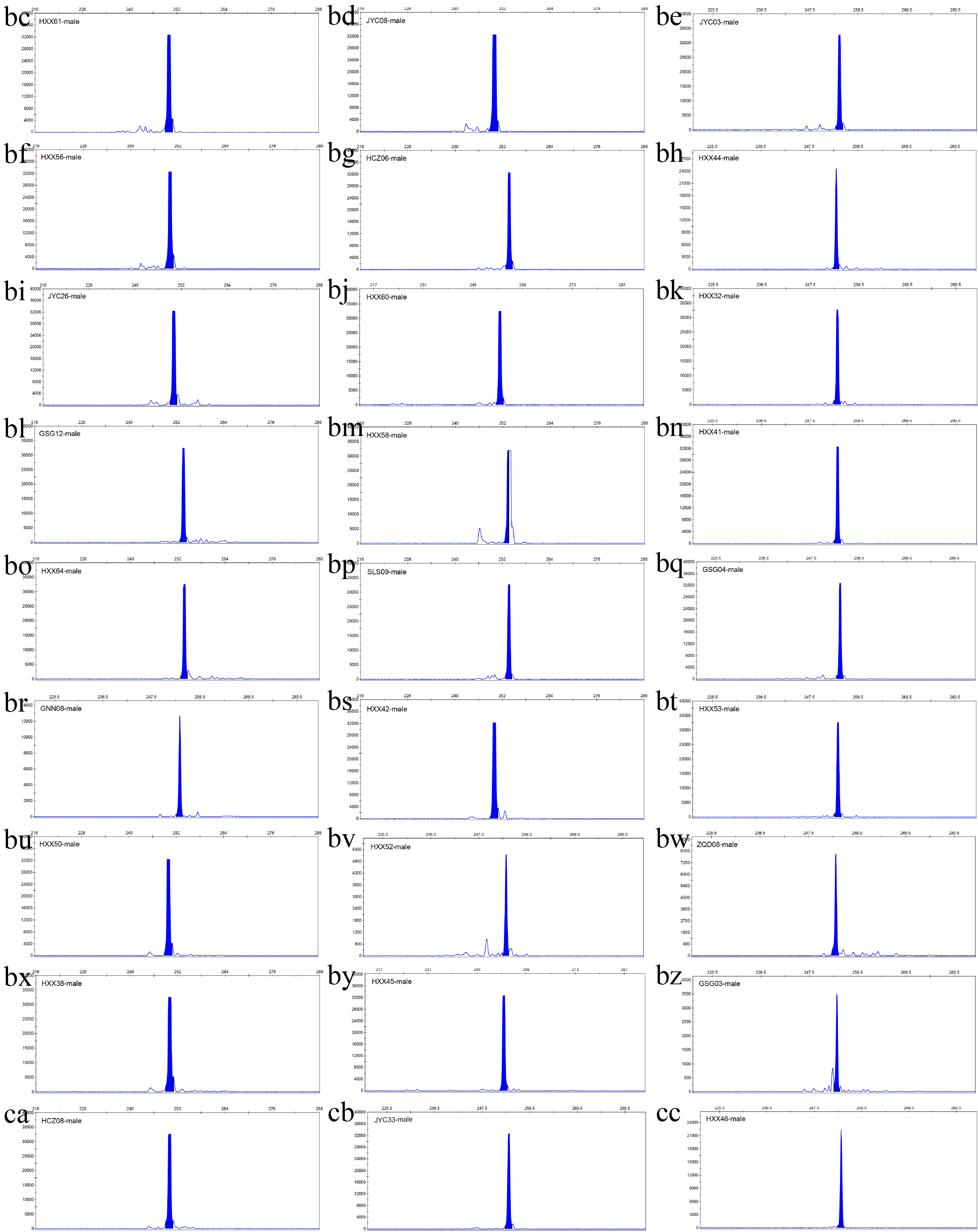


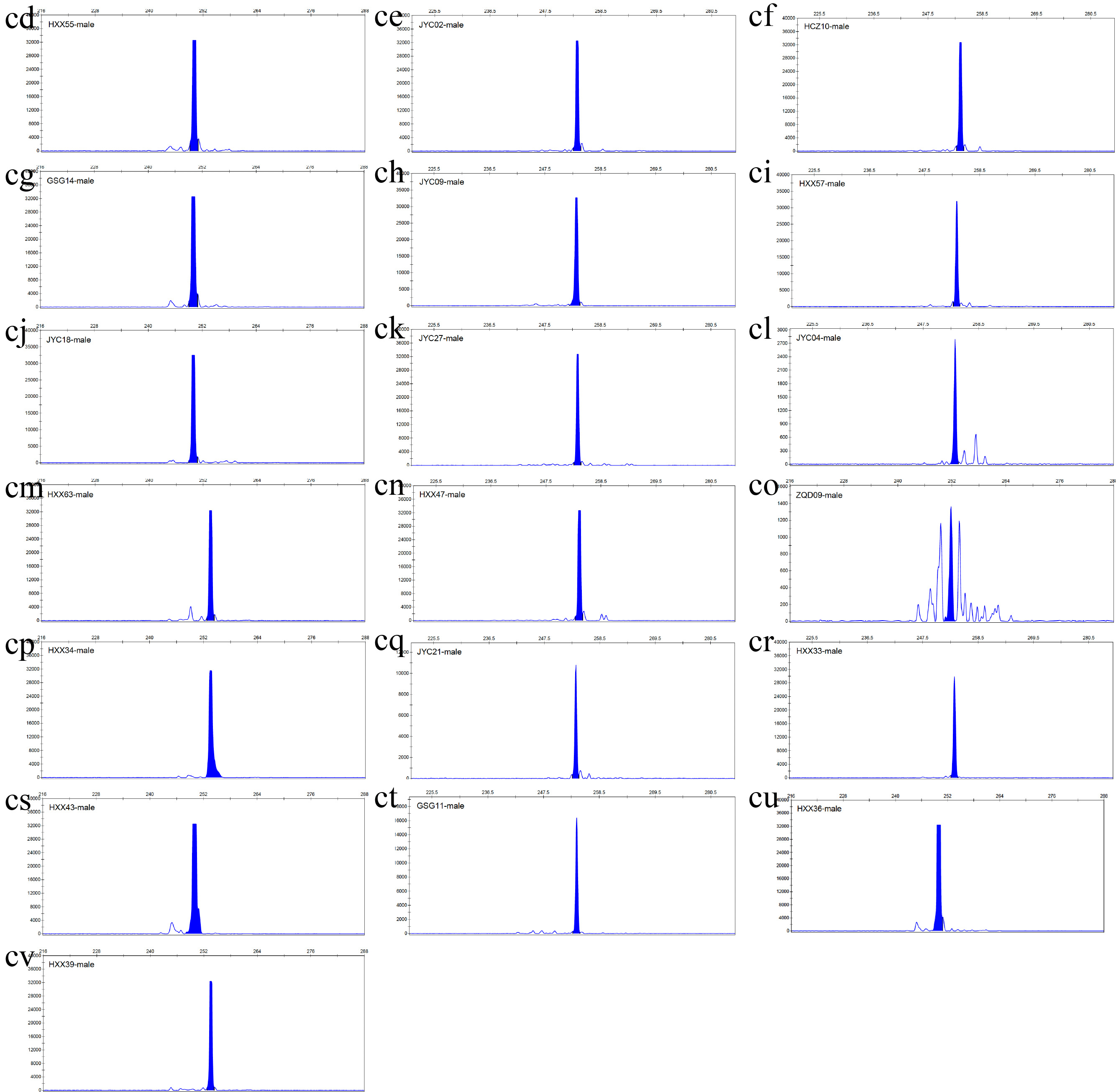


**Figure S20.** Amplification products of 50 female (a-ax) and 50 male (ay-cv) *V. montana* individuals using a capillary electrophoresis platform.


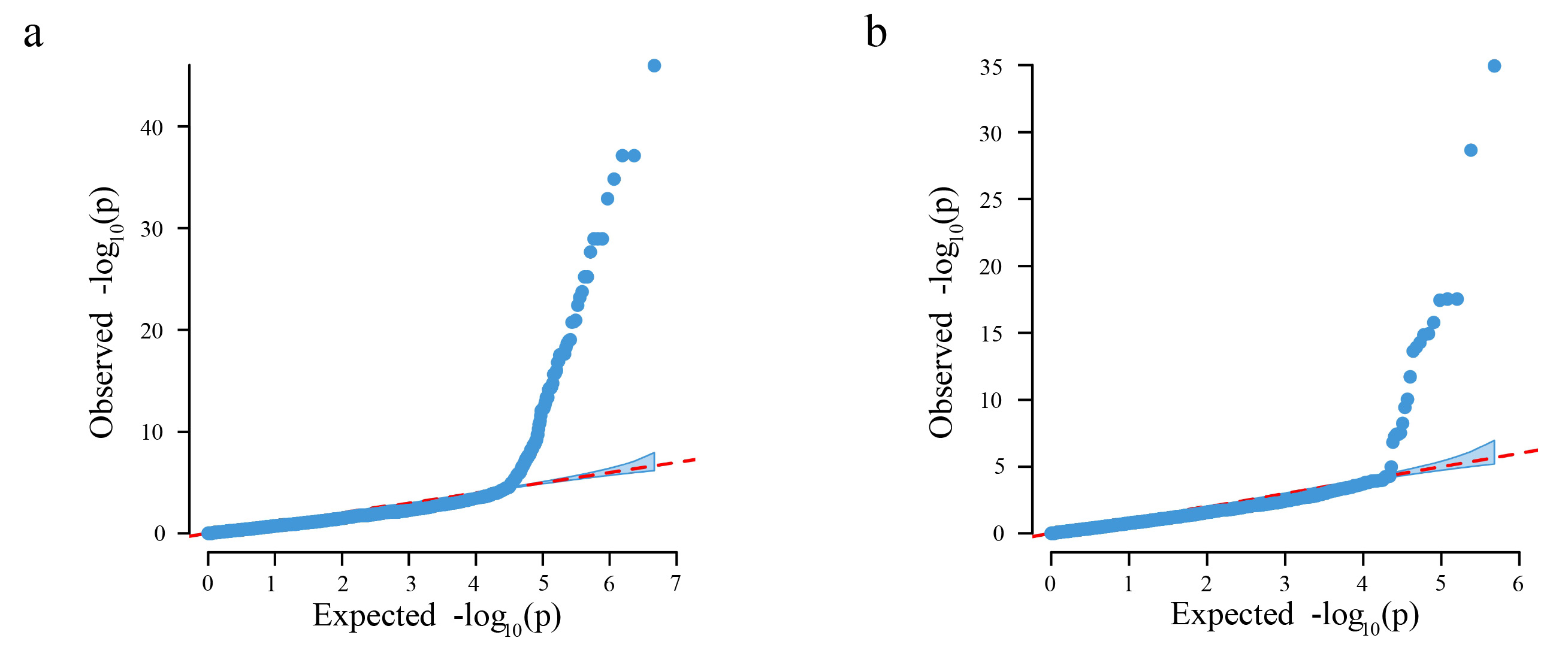


**Figure S21.** QQ plots of the results of GWAS analyses based on SNPs (a) and InDels (b).
